# Apoptotic Pathway as the Therapeutic Target for Anticancer Traditional Chinese Medicines

**DOI:** 10.3389/fphar.2019.00758

**Published:** 2019-07-12

**Authors:** Weixiao An, Honglin Lai, Yangyang Zhang, Minghua Liu, Xiukun Lin, Shousong Cao

**Affiliations:** ^1^Department of Pharmacology, School of Pharmacy, Southwest Medical University, Luzhou, China; ^2^Department of Pharmacy, Nanchong Central Hospital, Nanchong, China; ^3^Department of Pharmacy, Affliated Hospital of Traditional Chinese Medicine, Southwest Medical University, Luzhou, China

**Keywords:** apoptosis, cancer, cancer therapy, traditional Chinese medicine, cellular signaling pathway

## Abstract

Cancer is a leading cause of morbidity and mortality worldwide. Apoptosis is a process of programmed cell death and it plays a vital role in human development and tissue homeostasis. Mounting evidence indicates that apoptosis is closely related to the survival of cancer and it has emerged as a key target for the discovery and development of novel anticancer drugs. Various studies indicate that targeting the apoptotic signaling pathway by anticancer drugs is an important mechanism in cancer therapy. Therefore, numerous novel anticancer agents have been discovered and developed from traditional Chinese medicines (TCMs) by targeting the cellular apoptotic pathway of cancer cells and shown clinically beneficial effects in cancer therapy. This review aims to provide a comprehensive discussion for the role, pharmacology, related biology, and possible mechanism(s) of a number of important anticancer TCMs and their derivatives mainly targeting the cellular apoptotic pathway. It may have important clinical implications in cancer therapy.

## Introduction

**Figure 1:**
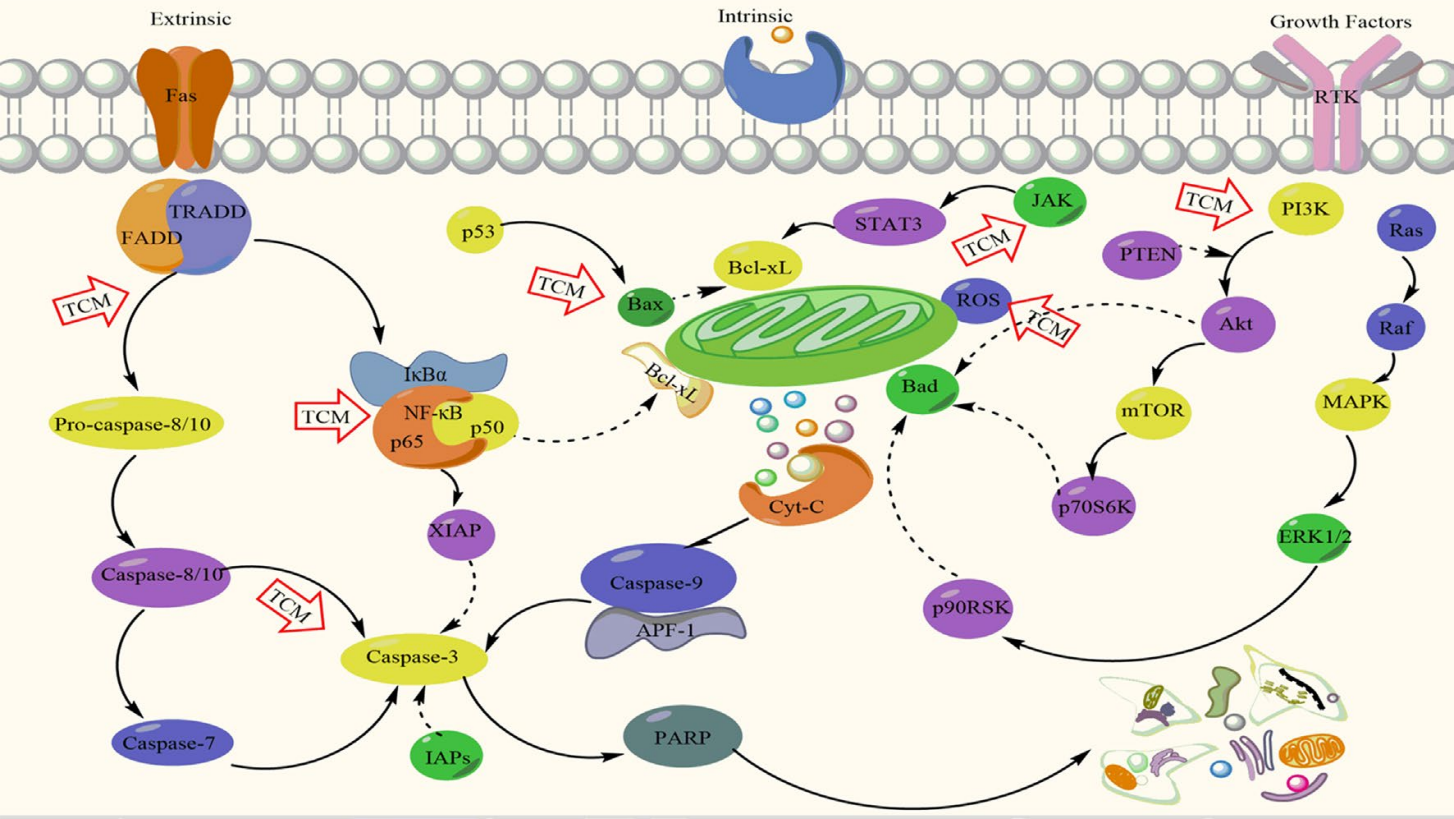
Mechanistic actions of apoptosis induction by traditional Chinese medicines (TCMs). Akt, protein kinase B; APF-1, ATP-dependent proteolysis factor 1; Bax, BCL2-associated X; Bcl, B cell lymphoma; Cyt c, cytochrome c; ERK1/2, extracellular regulated protein kinases; FADD, Fas associated death domain; IAPs, inhibitor of apoptosis proteins; IκBα, nuclear factor of kappa light polypeptide gene enhancer in B-cells inhibitor, alpha. JAK, Janus kinase; MAPK, mitogen-activated protein kinase; Mcl, myeloid cell leukemia sequence 1; mTOR, mammalian target of rapamycin; NF-κB, nuclear factor kappa B; PI3K, phosphatidylinositol-3 kinase; PARP, poly ADP-ribose polymerase; PTEN, phosphate and tension homology deleted on chromosome ten; Raf, rapidly accelerated fibrosarcoma; ROS, reactive oxygen species; RTK, receptor tyrosine kinase; STAT3, signal transducer and activator of transcription 3; TRADD, tumor necrosis factor (TNF) receptor associated death domain; XIAP, X-linked inhibitor of apoptosis protein.

Apoptosis is essential in the normal tissue development and homeostasis maintenance (Lockshin and Williams, 1965). Defect in apoptosis can cause various diseases such as autoimmunity, degenerative diseases, cancer, etc. (Hassan et al., 2014). Apoptosis is primarily initiated through two main signaling pathways: the death receptor (extrinsic) and mitochondria mediated (intrinsic) pathways. The two apoptotic pathways are linked with each other and one pathway can influence another pathway as illustrated in **Figure 1** (Igney and Krammer, 2002; Czabotar et al., 2014). However, an additional apoptotic pathway with growth factor receptors may also exist, which is closely related to the phosphatidylinositol-3 kinase (PI3K)–protein kinase B (Akt)–mammalian target of rapamycin (mTOR) and signal transducer and activator of transcription 3 (STAT3) pathways (Khan et al., 2015). The extrinsic apoptotic pathway is triggered by the interaction between Fas and Fas ligand or tumor necrosis factor (TNF)-related apoptosis-inducing ligand (TRAIL) and DR5 through activating the ligations of cell surface death receptors of TNF receptor superfamily (such as CD95L, TRAIL and TNF-α); the receptor-ligand activates caspase-8 or caspase-10 to stimulate the transcription factor nuclear factor-B (NF-κB) and mitogen-activated protein kinases (MAPKs) in adaptive, non-apoptotic signaling pathways associated with the regulation of developmental and inflammatory processes and then further to induce apoptosis (Sessler et al., 2013). In the intrinsic apoptotic pathway, apoptosis can be induced by release of cytochrome-c (Cyt-c) from the intramembrane into the cytosol for assembling of apoptosome (a caspase-activating multiprotein complex) further promoting caspase-9 activation (Luo et al., 1998; Korsmeyer et al., 2000; Green and Kroemer, 2004). Apoptosis can be also regulated by B-cell-lymphoma protein 2 (Bcl-2) family (Redza-Dutordoir and Averill-Bates, 2016). Both pathways can promote the initiator caspases for further activation of the effector caspases, which are the main executioner of apoptosis.

Traditional Chinese medicines (TCMs) have been widely used for the prevention and treatment of various diseases for over thousands of years in China and other Asian countries. Natural products and medicinal plants are a rich source of bioactive compounds for therapeutic agents and it has a long tradition for deriving drug substances from natural sources in medicine (Farver, 1996). In the early 1900s, 80% of medicines were obtained from natural sources and about 25% of prescribed drugs were originated from plants today worldwide (Aboul-Soud et al., 2015). Currently, 10% of the prescribed drugs are traditional Chinese herbal medicines in China (Li and Peng, 2013). Recently, TCMs have attracted substantial attention and already become an emerging field in cancer research. Anticancer TCMs are abound in the nature and easy to be obtained; more importantly, many of them have displayed significant antitumor efficacy with less toxic and side effects compared to traditional chemotherapeutic agents (Qiu and Jia, 2014). Furthermore, a study indicated that the combination of TCM and radiotherapy could reduce radiation-induced adverse effects and improve the quality of life in patients with non-small cell lung cancer (Cai et al., 2002). In this review, we mainly focus on the recent progress of some active anticancer TCMs and derived compounds with the primarily mechanistic actions *via* inducing apoptosis in cancer cells.

## Anticancer TCMs Through Activating Caspase Proteases

Caspases belong to the cysteinyl aspartate-specific proteases family, which is closely involved with apoptotic cell death. Dysregulation of caspases may cause various diseases in humans such as cancer and inflammatory disorders (Looi et al., 2013). Caspase family was categorized as the initiator caspases such as caspases-8, -9, and 10 and the effector caspases such as caspases-3, -6, and -7. The activation of caspases-3 and -7 is essential for inducing downstream DNA cleavage molecules, which is involved with both extrinsic and intrinsic apoptotic pathways (Mcllwain et al., 2013; Wu H. et al., 2014). The development of novel anticancer agents through the activation of caspases is one of the effective strategies in the treatment of cancer. Several active compounds and extracts derived from anticancer TCMs have been found to induce apoptosis by primarily targeting the activation of caspases for executing their anticancer activity including cordycepin, tetrandrine, the extracts of *Scutellaria barbata* D. Don, crocin, and the extracts of *Agrimonia pilosa* Ledeb.

*Cordyceps* species, also called as “冬虫夏草, winter worm summer grass” and a genus of ascomycete fungi, include approximately 400 species and some of them have been commonly used as tonics and stimulants for energy enhancement for a long time in China (Paterson, 2008). The preparations of *Cordyceps* particularly polysaccharides and secondary metabolites have the potential for the improvement of energy metabolism and are active against diabetes mellitus and cancer (Paterson, 2008; Kim et al., 2014). Cordycepin, a nucleoside analogue of 3-deoxyadenosine, is isolated and extracted from the fruiting bodies and fermentative solution of *Cordyceps militaris via* conventional methods such as pressurized, soxhlet, reflux, or ultrasound and microwave-assisted extraction (Ni et al., 2009). Previous studies showed that cordycepin was active against various cancer cells and the mechanistic action of anticancer activity was mainly *via* apoptosis induction (Chen et al., 2014). Cordycepin induces apoptosis primarily through activating caspases, although it targets multiple signaling pathways (Tian et al., 2015). Another study demonstrated that cordycepin promoted the activation of the pro-apoptotic factors of Bax and caspases-8, -9, and -3 and inhibited the anti-apoptotic factor of Bcl-2, and its effect on apoptosis induction was mainly *via* caspase-dependent pathways in human breast cancer MCF-7 and MDA-MB-231 cells (Wang D. et al., 2016). A recent study has found that encapsulated cordycepin in transferrin-conjugated liposomes exhibited anticancer activity *via* increasing the production of reactive oxygen species (ROS) and depolarization of the mitochondrial transmembrane in liver cancer HepG2 and PLC/PRF/5 cells (Bi et al., 2017).

*Stephania tetrandra* S. Moore is a commonly used TCM as diuretic, expectorant, and cathartic agent for over 400 years in China. Tetrandrine [(1b)-6,6′,7,12-tetramethoxy-2,2′-dimethyl-berbaman] is a bis-benzylisoquinoline (BBI) alkaloid isolated and extracted from the root of *St. tetrandra* S. Moore (Liu T. et al., 2016). Tetrandrine could induce apoptosis against multiple human cancer cell lines by activating caspases (Liu K. C. et al., 2017; Bhagya and Chandrashekar, 2018). For example, tetrandrine induced apoptosis *via* caspase activation and PARP cleavage in hepatic stellate cells (Bhagya and Chandrashekar, 2018). In addition, tetrandrine also induced apoptosis through caspase activation to increase ROS production against various human cancer cells including hepatic, prostate, cervical, breast, bladder, nasopharyngeal, glioma, and leukemia cancer cells (Liu K.C. et al., 2017; Bhagya and Chandrashekar, 2018). Encapsulated tetrandrine with microspheres, solid lipid liposomes, and nanoparticles were developed and investigated for improving the bioavailability of tetrandrine. The results showed that modified tetrandrine and paclitaxel nanoparticles could significantly inhibit cell proliferation and induce apoptosis *in vitro* and improve the bioavailability and enhance antitumor efficiency in an animal model of local implanted tumor *in vivo* against gastric cancer (Li X. et al., 2012; Zhang H. et al., 2016).

*S. barbata* D. Don (Labiatae) has been used as a TCM for “清热解毒, clearing away the heat and toxicity” in China for a long time (Zhang Y et al., 2017). Studies have shown that the extract of *S. barbata* D. Don exhibited obvious anticancer activity against mouse liver cancer H22 cells by inducing apoptosis *via* caspase-3 activation and Cyt-c release from cellular mitochondria (Dai et al., 2008). Another report also showed that the extract of *S. barbata* D. Don by chloroform at concentrations of 50–200 µg/ml was highly cytotoxic to human hepatocellular carcinoma Bel-7402 cells but was not toxic to normal liver cells (Yu et al., 2007). Mechanistic study further showed that its anticancer activity was through apoptosis induction by caspase-9 activation and Cyt-c release (Yu et al., 2007). In addition, the extract of *S. barbata* D. Don by methylene chloride also exhibited significant cytotoxicity against human leukemia U937 cells at the concentrations of 5–15 µg/ml *via* inducing apoptosis by activating caspases and altering the ratio of Bax/Bcl-2 (Cha et al., 2004). Yin et al. (2004) reported that *S. barbata* D. Don extract significantly inhibited cell proliferation by caspase-dependent apoptosis induction in lung cancer A549 cells. Xu H. et al. (2013) also showed that the ethanol extract of *S. barbata* D. Don at concentrations of 12.5–100 µg/ml significantly inhibited cell proliferation of various cancer cells and displayed synergistic anticancer effect when it was combined with a low dose of 5-fluorouracil. The study has also found that the anticancer effect of *S. barbata* D. Don extract was closely related to the apoptosis induction by caspases activation (Xu H. et al., 2013).

*Crocus sativus* L. (iridaceae) promotes the circulation of blood to avoid stasis and it may be effective for cancer treatment in TCM theory. Crocin belongs to carotenoid esters naturally occurring in *C. sativus* L. and generally exists in the flowers crocus and gardenia (Cormier et al., 1995). Reports have shown that crocin possesses multiple beneficial functions such as antioxidant, anticancer, and neural protection and crocin has been used for the treatment of cancer for a long time in China (Abdullaev et al., 2002; Papandreou et al., 2006; Ochiai et al., 2007; Yoshino et al., 2014). Crocin significantly inhibited cell proliferation and induced apoptosis against human osteosarcoma MG63 and OS732 cells at the concentrations of 0.5–4 mmol/L, and the effects of crocin on cytotoxicity and apoptosis were associated with the increased expression of caspases-3 and -8 (Li X. et al., 2013). Studies by Bakshi et al. (2010) showed that crocin had significant cytotoxicity by inducing apoptosis and cell cycle arrest at G1 phase in a concentration- and time-dependent manner in pancreatic cancer BxPC-3 cells. Crocin also exhibited cytotoxicity against human gastric cancer AGS cells by apoptosis induction through increasing caspase activation and Bax/Bcl-2 ratio (Hoshyar et al., 2013). Additional report also demonstrated that crocin was highly cytotoxic to human cervical cancer HeLa cells by markedly inducing apoptosis (Escribano et al., 1996). In addition, crocin showed potent cytotoxicity against colorectal cancer HT-29 and DHD/K12-PROb cells by inducing apoptosis *in vitro* and subcutaneous weekly injection of 400 mg/kg crocin increased the life span of rats with transplanted colon cancer without significant toxicity *in vivo* (García-Olmo et al., 1999). Furthermore, crocin also inhibited the cell growth and induced apoptosis and G0/G1 cell cycle arrest against leukemia HL-60 cells *in vitro*, and crocin at 6.25–25 mg/kg suppressed the growth of HL-60 tumor xenografts associated with the downregulation of Bcl-2 and upregulation of Bax increase in the tumor tissues *in vivo* (Sun et al., 2013).

**Figure 2:**
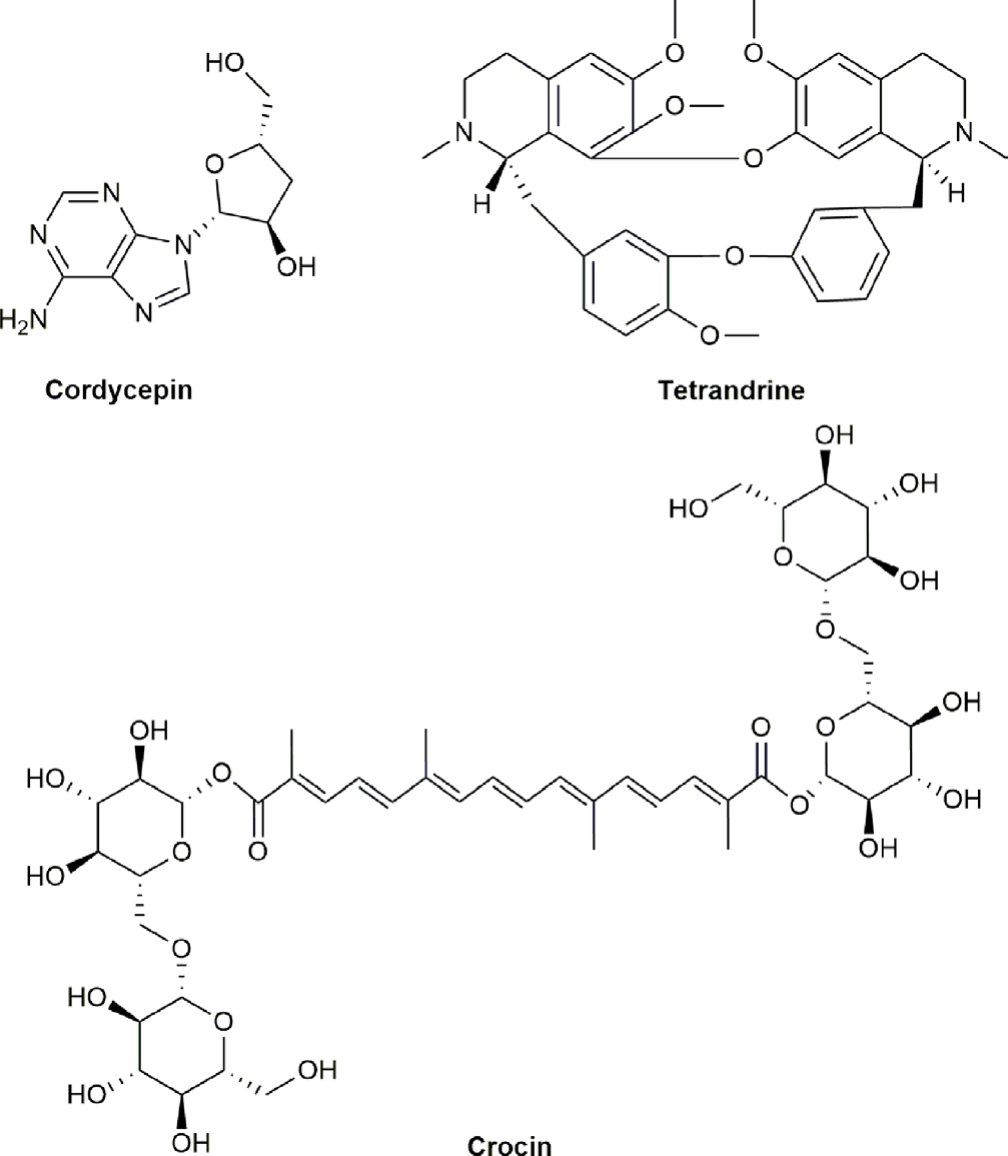
Chemical structures of active compounds derived from anticancer TCMs that primarily activate caspase proteases.

*A. pilosa* Ledeb. (Rosaceae) is a flowering plant and comprises many chemical components including agrimonolide, coumarin, fatty acid, flavonoids, isocoumarin, phenylpropanoids, tannins, and triterpenes (Liu W. J. et al., 2016). *A. pilosa* Ledeb. has been commonly utilized as a TCM for the treatment of abdominal pain, diarrhea, sore throat, headaches, heat stroke, and cancer in China and other Asian countries (Miyamoto et al., 1987; Kato et al., 2010; Ali et al., 2014). A study has showed that the extract of *A. pilosa* Ledeb. by ethanol significantly inhibited the cell proliferation and induced apoptosis at the concentrations of 50–400 µg/ml against liver cancer HepG2 cells associated with increasing the activity of caspases-3 and -9, PARP, Bcl-XL, BID, BIK, MCL-1, and XIAP (Nho et al., 2011). Studies also showed that the extract *of A. pilosa* Ledeb. significantly inhibited the growth of melanoma MM2 cells *in vitro* and prolonged the life span of mice bearing ascites tumor MM2 as well as solid tumors of human epithelial carcinoma MH134 and sarcoma Meth-A by oral, intravenous (i.v.), or intraperitoneal (i.p.) administration *in vivo* (Miyamoto et al., 1987). The chemical structures of active compounds derived from anticancer TCMs that activate caspase proteases are illustrated in **Figure 2**.

## Anticancer TCMs Through Affecting The Bcl-2/Bax Pathway

The Bcl-2/Bax pathway plays a key role in intrinsic apoptotic pathway, which is dependent on the ratio of Bcl-2 and Bax in the mitochondria. Specifically, Bcl-2 protein can block the efflux of Cyt-c from cytosol to mitochondria to prevent caspase activation, then further inhibiting apoptosis. Bax protein acts as apoptosis promoter while Bcl-2 protein as apoptosis suppressor (Gross et al., 1999). The Bcl-2 family consists of pro-survival members such as Bcl-2, Bcl-xL, Bcl-W, Bcl-B, MCL-1, and A1/BFL-1 and pro-apoptotic members such as Bax, BAK, and possibly BOK; all the family members share the common domains of Bcl-2 homology (BH) (Echeverry et al., 2013). The Bcl-2 family controls the integrity of the mitochondrial outer membrane (MOM) to regulate the intrinsic pathway for release of apoptogenic molecules; therefore, control of the integrity of MOM mediates the response to most cytotoxic therapies (Cory et al., 2016). Previous reports showed that anti-apoptotic Bcl-xL protein could induce chemoresistance and inhibition of this protein revised drug resistance in melanoma cells (Hata et al., 2015). Therefore, balance of Bcl-2 family members and the ratio of Bcl-2/Bax determine whether the cell is undergoing apoptosis or is surviving. Many TCMs have been proved to generate cytotoxicity and induce apoptosis *via* the regulation of the balance of Bcl-2 family and/or the ratio of Bcl-2/Bax in various cancer cells. Here, we review some anticancer TCMs and derived active compounds that induce apoptosis primarily *via* targeting the Bcl-2/Bax pathway such as *Prunella vulgaris* (PV), hyperoside, *Rabdosia rubescens* (RR) Oridonin, Solamargine (SM), the extracts of *Solanum lyratum Thunb*, Huaier and its extracts, *Hedyotis diffusa* Willd and its extracts, Gambogic acid, and Telekin.

PV, an edible plant and TCM, has potent biological activity including anti-proliferation and inducing apoptosis against cancer cells (Zhang et al., 2006; Feng et al., 2010). PV induced apoptosis by alteration of the Bcl-2/Bax ratio and activation of caspase-3 in thyroid carcinoma cells (Yin et al., 2017). Furthermore, the combination of PV and taxane could increase the anticancer efficacy and reduce taxane-induced toxicity in patients with breast cancer, thus preventing the progression of disease clinically (Zhao et al., 2018). Hyperoside, the major pharmacologically active component of PV, could significantly inhibit the proliferation and induce apoptosis in lung cancer A549 cells (Yang Y. et al., 2017).

RR is a medical herb plant and has multiple biological and pharmaceutical activities including anti-inflammation, antibacteria, anticancer, and neuroprotection (Bai et al., 2010; Li D. et al., 2016). RR could inhibit the cell growth and induce apoptosis in human breast cancer MDA-MB231 cells (Li D. et al., 2016). RR and its extracts are clinically effective for the treatment of patients with different types of cancer such as esophageal, gastric, liver, and breast cancers by alleviating syndromes, reducing tumor burden, suppressing disease progress, and prolonging survival in China (Wang and Wang, 1994). The fractions of RR extracts by chloroform and ethyl acetate could reverse the multidrug resistance (MDR) in human breast cancer MCF-7/Adr cells (Li F. et al., 2013). Oridonin, a diterpenoid extract from RR, is the most important active ingredient for its anticancer efficacy (Li D. et al., 2016). Oridonin remarkably induced apoptosis in gastric, breast, and colorectal cancer cells (Li D. et al., 2016; He et al., 2017; Qi et al., 2017a; Qi et al., 2017b). Oridonin significantly inhibited the cell growth and induced apoptosis against human gastric cancer SGC-790 cells *via* alteration of the Bcl-2/Bax ratio by downregulation of the antiapoptotic Bcl-2 protein and upregulation of the proapoptotic Bax protein (Gao et al., 2016). Further studies showed that oridonin at the concentrations of 12.5–100 μM significantly induced apoptosis against liver cancer MHCC97-H cells and the effect was related to decrease of the Bcl-2/Bax ratio and increase of the activity of caspases-3, -9, and Cyt-c (Zhu et al., 2013). Additional studies also showed that oridonin could inhibit cell growth and induce apoptosis in cervical cancer HeLa, gallbladder cancer SGC996 and NOZ, and leukemia K562 cells *via* alteration of the Bcl-2/Bax ratio with decreased expression of Bcl-2 and increased expression of Bax in mitochondria (Zhang et al., 2004; Liu et al., 2005; Bao et al., 2014). Oridonin also synergistically increased the cytotoxicity of Nutlin-3 [an inhibitor of murine double minute 2 (Mdm2)] against wild-type p53 osteosarcoma U2OS cells by enhancing cellular apoptosis *via* increasing the level of proapoptotic factor Bim and decreasing the levels of antiapoptotic factors Bcl-2 and Bcl-xl (Wang X. H. et al., 2017). Furthermore, oridonin also significantly increased the cytotoxicity and/or reversed drug resistance of various anticancer drugs such as cisplatin, gemcitabine, and lentinan by downregulation of Bcl2 expression and upregulation of Bax expression in multiple human cancer cell lines including ovarian, pancreatic, and liver cancers (Liu D. L. et al., 2014; Ma et al., 2016; Xu T. et al., 2017).

*Solanum incanum* is a spreading perennial plant belonging to the Solanacea family. *So. incanum* has the function of pain-relieving and is widely used as a TCM in China. Previous report revealed that SM, a steroidal alkaloid glycoside isolated and extracted from *So. incanum*, significantly induced apoptosis and G2/M cell cycle arrest with alteration of the Bcl-2/Bax ratio by downregulating antiapoptotic Bcl-2 and upregulating apoptotic Bax and caspases-3 and -9 in liver cancer SMMC7721 and HepG2 cells (Xie et al., 2015). A study also showed that SM significantly induced apoptosis by inhibiting Bcl-2 protein and promoting Bax protein in human cholangiocarcinoma QBC939 cells (Zhang X. et al., 2018). Additional studies further demonstrated that SM could selectively inhibit the proliferation of primary and metastatic melanoma cells WM115 and WM239 with minimum cytotoxicity to normal WM35 cells (Al Sinani et al., 2016). In addition, SM could increase the therapeutic efficacy of commonly used therapeutic drugs such as cisplatin, epirubicin, and trastuzumab against human breast and lung cancer cells (Shiu et al., 2007; Liang et al., 2008). Therefore, SM may be developed as a novel therapeutic agent for cancer therapy used alone or in combination with other therapeutic agents clinically.

*So. lyratum Thunb* is a medical plant also belonging to the Solanacea family. *So. lyratum Thunb* is one of the most popular TCMs as “Hedrba Solina Lyrati” for immune boost and has been widely used medicinally for allergy treatment in China (Kang et al., 1997). Study has shown that the extract of *So. lyratum Thunb* significantly inhibited the cell growth and induced apoptosis in murine leukemia WEHI-3 cells *in vitro* and exhibited marked antitumor activity against WEHI-3 allografted tumors *in vivo* (Yang et al., 2012). The ethanol extract of *So. lyratum Thunb* could significantly produce cytotoxicity and induce apoptosis *via* alteration of the Bcl-2/bax ratio by downregulating the level of Bcl-2 protein while upregulating the level of Bax protein in human osteosarcoma U-2 OS cells (Lin et al., 2013). The total saponins isolated and extracted from *So. lyratum Thunb* exhibited obvious cytotoxicity *via* induction of apoptosis by decreasing Bcl-2 expression while increasing Bax expression in human cervical cancer Hela cells (Liu H. R. et al., 2008). Furthermore, the extract of *So. lyratum Thunb* displayed significant antitumor activity and increased the survival of mice bearing Lewis lung carcinoma and S180 sarcoma (Guan et al., 2013; Xiao et al., 2013).

*Trametes robiniophila* Murr. (huaier) is a mushroom with the function of “衡体解痢, homeostasis and relieving dysentery” and has been commonly used as a TCM for over 1,600 years in China (Li et al., 2007). Report has shown the anti-angiogenic and anticancer potential of *T. robiniophila* Murr. (Wang et al., 2012). Several studies have shown that *T. robiniophila* Murr. could inhibit the cell growth and induce apoptosis in human lung cancer A549, renal cancer 786-O, and prostate cancer PC3 cells (Wu T. et al., 2014; Wei et al., 2018; Yang et al., 2018). Multiple studies have demonstrated that the aqueous extract of *T. robiniophila* Murr. was able to inhibit the proliferation and induce apoptosis by targeting the ratio of Bcl-2/Bax *via* inhibiting the antiapoptotic Bcl-2 protein and promoting proapoptotic Bax protein as well as other cellular signaling pathways in breast cancer MCF-7 and MDA-MB-231, gastric cancer GC, ovary cancer SKOV3, fibrosarcoma HT1080, and melanoma A875 cells (Zhang et al., 2010; Yan et al., 2013; Zhang F. et al., 2013; Cui et al., 2015; Xu Z. et al., 2017). In addition, *T. robiniophila* Murr. could also significantly increase the anticancer efficacy of clinically commonly used therapeutic drugs including paclitaxel, cisplatin, and rapamycin against human breast, liver, and lung cancer cells *in vitro* and animal models of tumor xenografts *in vivo* (Hu Z. et al., 2016; Yang L. et al., 2017). Furthermore, *T. robiniophila* Murr. also could sensitize radiotherapy against breast cancer MDA-MB-468 and MCF-7 cells (Ding et al., 2016).

*H. diffusa* Willd belongs to the family of Rubiaceae and is a popular TCM widely used for the treatment of fevers, coughs, asthma, jaundice, impure blood, urinary tract infections, acute appendicitis, biliousness, and digestive tract cancer internally, as well as the treatment of snake bites, boils, abscesses, and severe bruising externally in China (Niu and Meng, 2013). It is a major component in the formulations of several medicines used for cancer treatment in China (Rui and Yining, 2002). *H. diffusa* Willd markedly inhibited the cell growth and induced apoptosis *via* significantly downregulating the mRNA levels of Bcl-2, IL-6, and VEGF in myeloma RPMI 8226 cells (Zhang X. et al., 2012). *H. diffusa* Willd also obviously inhibited the cell proliferation and induced apoptosis by decreasing the expression of Bcl-2 and increasing the expressions of caspases-9 and -3 in ovarian cancer A2780 cells (Zhang L. et al., 2016). The ethanol extract of *H. diffusa* Willd at 0.5–5 mg/ml could induce apoptosis *via* mitochondria-mediated apoptotic pathway by alteration of the ratio of Bcl-2/Bax with downregulating antiapoptotic Bcl-2, upregulating proapoptotic Bax, and activating caspases-3 and -9 in colon cancer HT-29 cells (Lin et al., 2010). The chloroform extract of *H. diffusa* Willd could significantly inhibit the cell growth and induced apoptosis by downregulating the expressions of antiapoptotic Bcl-2 and survivin and upregulating the expression of proapoptotic Bcl-2-X in colorectal cancer SW620 cells (Yan et al., 2017). Moreover, the combination of *H. diffusa* Willd and clinically commonly used chemotherapeutic drug 5-flurourical could significantly inhibit the cell growth, induce apoptosis, and revise drug resistance by decreasing Bcl-2 expression and increasing Bax expression in colorectal cancer HCT-8 and liver cancer HepG2 cells (Chen et al., 2012; Li et al., 2018b). Clinical study further showed that the combination of *H. diffusa* Willd with chemotherapeutic agents could significantly reduce drug-induced side effects and prolong the survival of patients with advanced liver cancer or esophageal cancer (Liu and Yao, 2004).

*Garcinia hanburyi* Hook. f. (Clusiaceae) is an evergreen tree and its orange resin called gamboge has been used for centuries as a TCM for detoxification, parasite-killing, and stopping bleeding (Wu Z. Q. et al., 2004). Gambogic acid is the key active component isolated and extracted from gamboge and exhibited significant anticancer effect against multiple human cancer cells and tumors such as lung, breast, gastric, liver, prostate, and pancreatic cancers, and leukemia and lymphoma *in vitro* and/or *in vivo*, while the primary mechanism may be due to inducing mitochondria-dependent apoptosis by alteration of the Bcl-2/Bax ratio (Wu Z. Q. et al., 2004; Mu et al., 2010; Xu J. et al., 2013; Duan et al., 2014b; Sun et al., 2018; Youns et al., 2018). Gambogic acid could also enhance the effects of chemotherapeutic drugs such as 5-fluorouracil, doxorubicin, oxaliplatin, and proteasome inhibitor (MG132 or MG262) as well as radiotherapy on cytotoxicity and apoptosis against human colorectal, breast, gastric, esophageal, liver cancers, and leukemia cells (Huang et al., 2011; Zou et al., 2012; Wang S. et al., 2015; Yang et al., 2016; Wei et al., 2017). Furthermore, a previous study with clinical trials of cancer patients showed that gambogic acid achieved clinical remission and benefit rates of 14.29% and 76.19%, respectively (Zhou and Wang, 2007).

**Figure 3:**
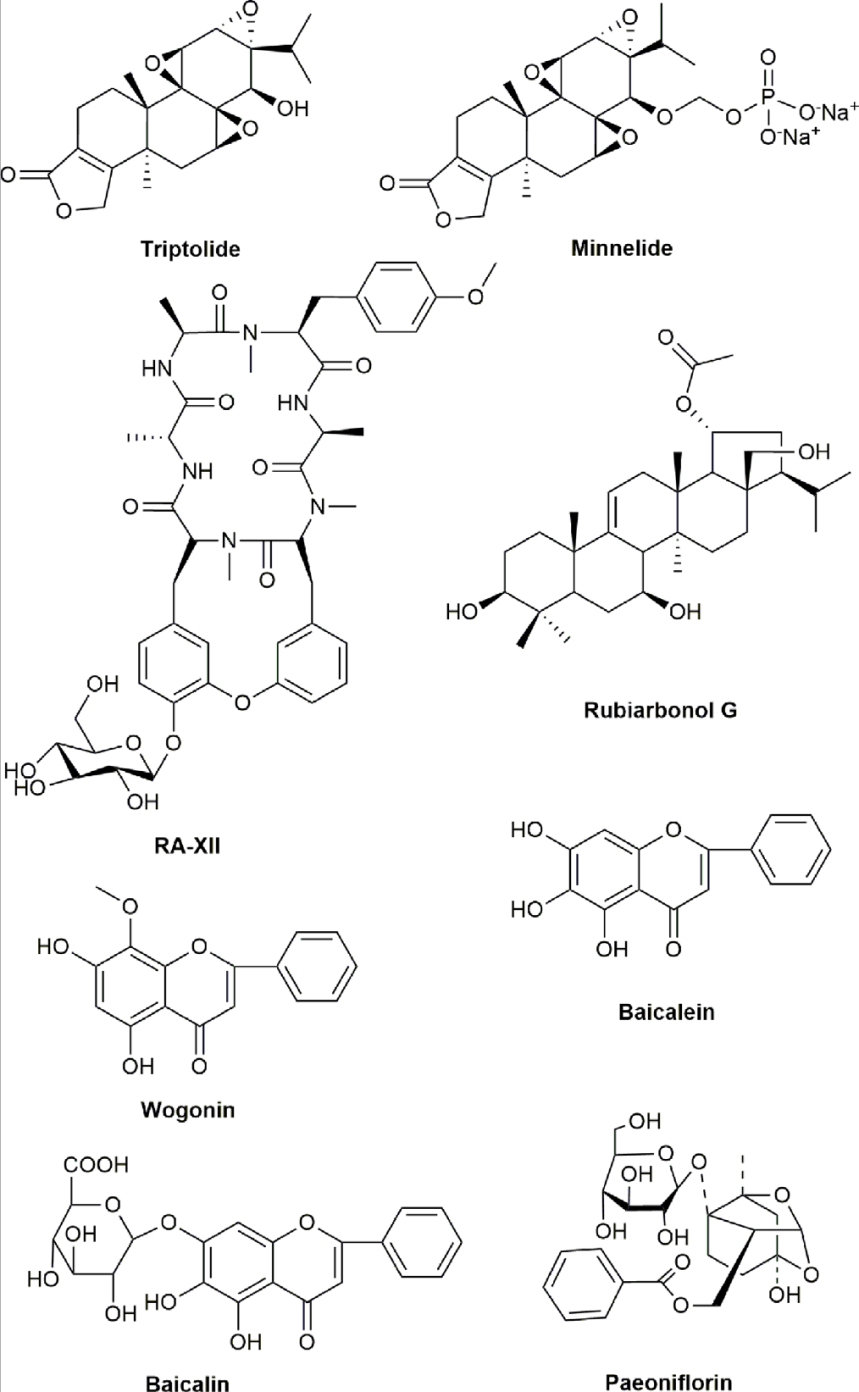
Chemical structures of active compounds from anticancer TCMs that primarily affect Bcl-2/Bax signaling pathway.

*Carpesium divaricatum* is a TCM with anti-inflammatory, analgesic, vermifugic, hemostatic, and detoxificative functions and widely used for the treatment of bruise, clods, fever, and snakebite (Zhang J. P. et al., 2015). Telekin was isolated and extracted from *C. divaricatum* and strongly inhibited the growth of various cancer cells (Kim et al., 1997; Paukku et al., 2009). Studies have shown that telekin at 2.5–10 µmol/L could significantly inhibit the cell proliferation by inducing cellular apoptosis *via* decreasing the expressions of Bcl-2 and Apaf-1, increasing the expression of Bax, releasing Cyt-c, and activating caspases-9 and -3 in liver cancer HepG2 cells (Zheng B. et al., 2013). Additional studies also showed that Telekin displayed potent cytotoxicity and induced apoptosis against multiple cancer cells (Li et al., 2014; Wang X. et al., 2015). The chemical structures of active compounds from anticancer TCMs that affect Bcl-2/Bax signaling pathway are illustrated in **Figure 3**.

## Anticancer TCMs Through Inhibiting The NF-κB Pathway

NF-κB is a family of transcription factors with five Rel-domain (RelA, RelB, c-Rel, NF-κB1/p50, and NF-κB2/p52) and plays a key role in tumorigenesis and chemoresistance (Kunnumakkara et al., 2012; Zeligs et al., 2016). The aberrant expression of NF-κB had been detected in most of cancer cells and the inhibitory κB (IκB) kinases (IKKs) are well recognized as key regulators of the NF-κB pathway (Karin and Ben-Neriah, 2000). The activation of NF-κB is closely related to chemotherapy-induced resistance and inhibition of NF-κB could sensitize cancer cell response to chemotherapeutic agents, therefore improving the anticancer efficacy of various anticancer agents in cancer therapy (Hassan et al., 2014; Naumann and Sokolova, 2017). Study has showed that the pro-apoptotic activity by inhibiting NF-kB was through either cell cycle inhibition or MAPK pathway downregulation (Xiao et al., 2016). Here, we review some typical active compounds derived from anticancer TCMs, which induce apoptosis primarily through inhibiting NF-κB signaling pathways such as triptolide (TP), minnelide, RA-XII, rubiarbonol G, wogonin, baicalein, baicalin, the extracts of cortex lycii radices, and paeoniflorin.

*Tripterygium wilfordii* Hook. f. (Celastraceae) has been commonly used as a TCM for the treatment of rheumatoid arthritis for a long time in China and TP is a principal diterpenoid triepoxide isolated and extracted from *T. wilfordii* Hook. f. (Lin et al., 2001). Although TP as a TCM is mainly used for the treatment of rheumatoid arthritis, it also attracted substantial attention in cancer research recently, and it has the potential to be developed as a novel anticancer agent. Study of the relationship between chemical structure and activity of TP showed that the five-membered unsaturated lactone D ring is crucial for its anticancer effect and c19 site is highly sensitive to polarity (Xu et al., 2014). Studies showed that TP displayed a broad spectrum and potent anticancer activity *via* inducing apoptosis in breast cancer, melanoma, and glioma cells (Cheng et al., 2016; Jao et al., 2016). In addition, the mechanistic studies showed that its anticancer effect is almost *via* interfering with the NF-kB signal pathway. Furthermore, TP binds to activate p38α and extracellular signal-regulated kinase 1/2 (ERK1/2) for phosphorylation and stabilization of p53; subsequently, p53 competes with IκBα for IKKβ binding to block the phosphorylation of IκBα and nuclear translocation of NF-κB in lung cancer cells (Zheng et al., 2017). The combination of TP with chemotherapeutic agents could overcome drug resistance and increase anticancer activity through the inhibition of NF-κB (Jiang N. et al., 2016). For example, the combination of TP and a novel heat shock protein 90 inhibitor BIIB021 exhibited synergistic anticancer effect against thyroid carcinoma cells *via* inhibiting NF-κB signal pathways (Kim et al., 2016). The combination of TP and sodium cantharidinate exhibited a synergistically cytotoxic effect against human hepatoma 7721 cells *via* increasing capase-3 activity and suppressing NF-κB (Zhou et al., 2016). Furthermore, the combined treatment of TP and ionizing radiation also exhibited synergistic effects of antiangiogenesis and anticancer against nasopharyngeal carcinoma *via* downregulating NF-κB p65 phosphorylation (Zhang W. et al., 2016). Minnelide, a TP analog, was designed to improve the solubility and anticancer efficacy (Chugh et al., 2012). Several studies have shown that minnelide significantly inhibited the growth and induced apoptosis against pancreatic cancer cells *in vitro* and tumors *in vivo* (Chugh et al., 2012), non-small cell lung carcinoma (Rousalova et al., 2013), and osteosarcoma (Banerjee et al., 2013), indicating that it could be potentially developed as an effective anticancer drug clinically.

*Rubia yunnanensis* (Franch) is a medicinal plant and its root (named as “Xiaohongshen”) has been used for the treatment of anemia, hematemesis, menoxenia, rheumatism, contusion, tuberculosis, and lipoma in China for thousands of years (Lan, 1978). The active ingredients were identified from *R. yunnanensis* Diels and displayed anticancer activity, and five (nos. 2, 7, 8, 9, and 10) of the identified compounds significantly inhibited the activation of NF-κB induced by TNF-α in human embryonic kidney (HEK) 293 cells with IC_50_ values of 35.07, 0.03, 1.69, 12.64, and 1.18 μM, respectively (Fan et al., 2010). RA-XII, one of the major active ingredients of *R. yunnanensis*, showed the effects of anti-oxidant and anti-inflammation on lipopolysaccharide (LPS) induced acute renal injury by inhibiting NF-κB (An and Shang, 2018). Another major active ingredient rubiarbonol G showed potent cytotoxicity against seven human cancer cells *via* inducing NF-κB and JNK mediated apoptosis and G0/G1 cell cycle arrest (Zeng et al., 2018).

Some flavonoid compounds from plants and fruits have the effect on downregulation of NF-κB. *Scutellaria baicalensis* Georgi (Labiatae) is a popular TCM and used for “清热泻火, heat-clearing and fire-purging” as described in the “Compendium of Materia Medica” for the treatment of sore throat symptoms such as swelling and pain. Three active flavones were isolated and extracted from *Sc. baicalensis* Georgi as wogonin, baicalein, and baicalin, and all of them exhibited anticancer activity against several cancer cells *via* inhibiting NF-κB activity (Li-Weber, 2009).

Cortex lycii radicis, the root bark of *Lycium chinense* Miller (Solanaceae), has been used as a TCM for the treatment of inflammatory symptoms in China. Studies have reported that six purified compounds isolated and extracted from cortex lycii radicis were evaluated and two isolated phenolic amides could inhibit NF-κB induced by TNF-α; trans-*N*-caffeoyltyramine is the key compound responsible for NF-κB inhibition (Xie L. W. et al., 2014). Further study on the structure–activity relationship indicated that the effect of trans-*N*-caffeoyltyramine on NF-κB inhibition may be related to the Michael acceptor-type structure (α,β-unsaturated carbonyl group) (Xie L. W. et al., 2014). Another report also showed that a crude extract of cortex lycii radicis strongly inhibited the proliferation and migration of human glioblastoma cells *in vitro* and significantly induced apoptosis associated with increased proapoptotic factors caspase-3 and Bax, and decreased antiapoptotic factor Bcl-2 in glioblastoma tumors *in vivo* (Wang Q. et al., 2016).

**Figure 4:**
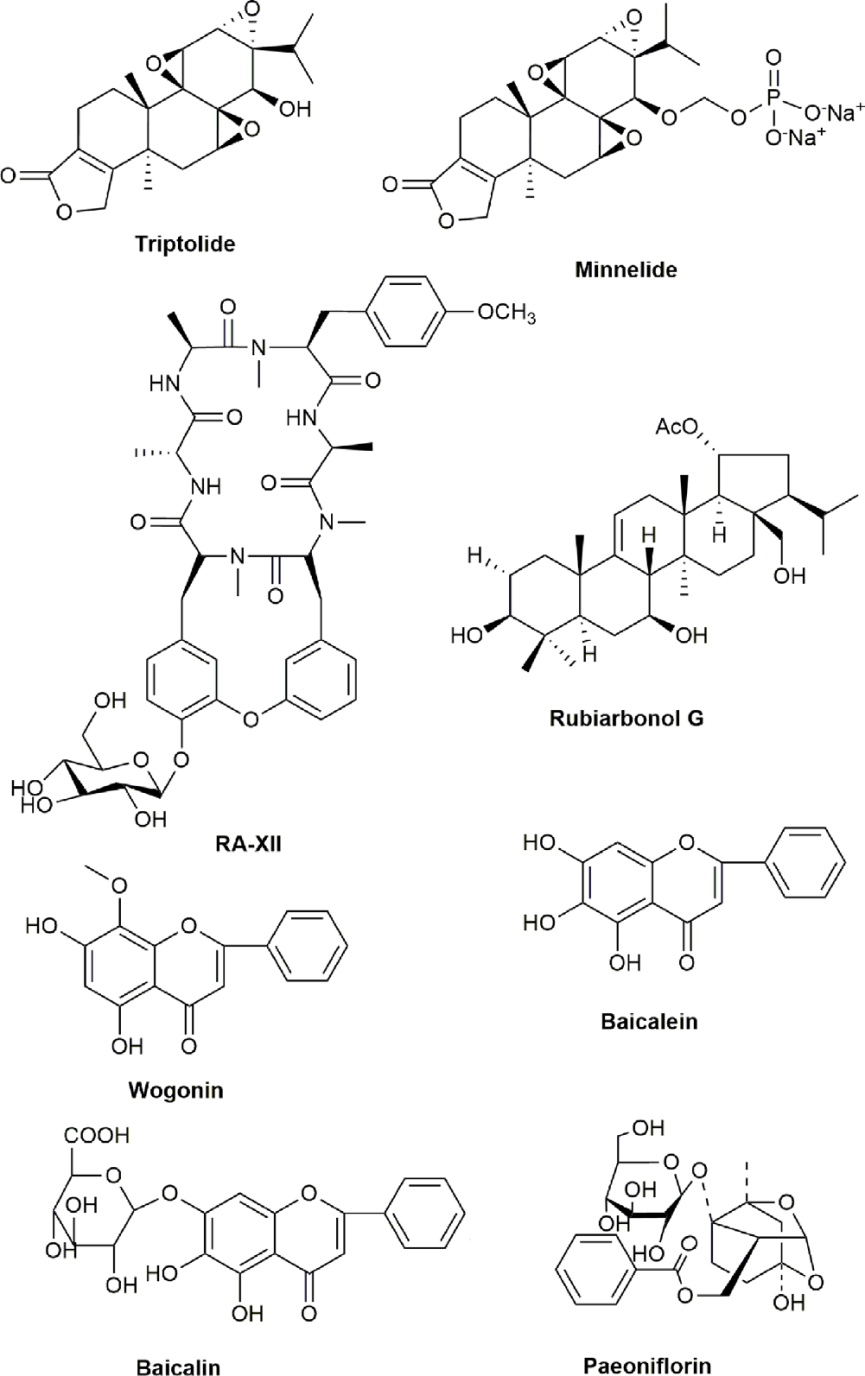
Chemical structures of active compounds derived from anticancer TCMs that primarily inhibit NF-κB signaling pathway.

*Paeonia lactiflora* Pall (Ranunculaceae) is a flowering plant and well-known TCM used for the treatment of rheumatoid arthritis in China for over a thousand years (He and Dai, 2011). A study has shown that paeoniflorin, a major bioactive ingredient of *P. lactiflora* Pall root, inhibited transcriptional activity of NF-κB stimulated by LPS in human colon cancer HT-29 cells (Zhang et al., 2014). The preparation of *Nigella sativa* (belonging to Ranunculaceae family) oil had potent inhibitory effect on cancer cells and its cytotoxicity was through the inhibition of NF-κB signaling pathway (Agbaria et al., 2015). Recent report also showed that the extract of *N. sativa* could produce cytotoxicity and induced apoptosis against human lymphoma U937 cells, but is not toxic to normal human vascular endothelial ECV304 cells (Arslan et al., 2017). The chemical structures of active compounds from anticancer TCMs that mainly inhibit NF-κB signaling pathway are illustrated in **Figure 4**.

## Anticancer TCMs Through Activating Reactive Oxygen Species

ROS is oxygen-derived species including superoxide, hydrogen peroxide singlet oxygen, and hydroxyl radical and plays a crucial role in cancer development and apoptosis (Simon et al., 2000; Waris and Ahsan, 2006; Hayyan et al., 2016). It is well known that anticancer agents can generate ROS to mainly accumulate ROS in the mitochondria of cancer cells for activating apoptotic signaling pathways including PI3K/Akt, MAPKs, NF-κB, nuclear factor (erythroidderived2)-like 2 (Nrf2)/Kelch like-ECH-associated protein 1 (Keap1), and the tumor suppressor p53 and finally induce cancer cell damage and death (Halliwell, 2011; Jeong and Joo, 2016; Redza-Dutordoir and Averill-Bates, 2016). Therefore, discovery and development of anticancer agents for overproduction of ROS in cancer cells is a good strategy in cancer therapy. Interestingly, some active compounds derived from anticancer TCMs such as curcumin and its analogues, shikonin, polysaccharides of *Pleurotus abalonus*, jaridonin, longikaurin A, physalin A, and physalin B, exhibit the ability to increase the level of ROS and induce apoptosis in cancer cells.

Curcumin, [(1E,6E)-1,7-bis(4-hydroxy-3methoxyphenol)-1,6-heptadiene-3,5-dione, C_21_H_20_O_6,_ MW: 368.37] is a natural compound extracted and purified from the root of the *Curcuma longa* Linn (a member of the family zingiberaceae) as a food additive for over centuries (Maheshwari et al., 2006). Curcumin as a TCM has multiple biologic activities including anti-inflammation, anti-angiogenesis, anti-oxidation, and cancer prevention (Rahmani et al., 2014). Studies have shown that curcumin could induce apoptosis *via* overproduction of ROS in human osteosarcoma MG63, skin cancer COLO-16, and lung cancer A549 cells (Chang et al., 2014; Jeong and Joo, 2016). Other studies also showed that curcumin induced cytotoxicity and apoptosis *via* overproduction of ROS in lung cancer A549 cells (Kaushik et al., 2012). Furthermore, curcumin could potentiate the anticancer efficacy of cisplatin via ROS-mediated pathway in bladder cancer 253J-Bv and T24 cells (Park et al., 2016). Although curcumin has anticancer potential, its clinical application in cancer therapy is limited because of its poor bioavailability and pharmacokinetic profiles. Therefore, several curcumin derivatives with better bioavailability and stability have been designed and developed as novel anticancer agents such as B63, B19, EF24, WZ26, WZ35, L48H37, and MAC CA10 (Zheng A. et al., 2014; Chen W. et al., 2016; He et al., 2016; Zou et al., 2016; Chen et al., 2017; Feng et al., 2017; Rajamanickam et al., 2017; Zhang J. et al., 2017; Wang L. et al., 2018). B63, a mono-carbonyl analogue of curcumin, exhibited potent cytotoxicity by significantly inducing apoptosis and necrosis in colon cancer SW620 cells *in vitro* and markedly inhibited the growth of SW620 tumor xenografts *in vivo*; the anticancer effect of B63 was mediated by overproduction of ROS in cancer cells (Zheng A. et al., 2014). Studies showed that another curcumin derivative B19 induced cell cycle arrest and apoptosis by activating ROS-mediated endoplasmic reticulum (ER) stress against human gastric cancer SGC-7901, BGC-823, and KATO III cells *in vitro* and inhibited tumor growth of SGC-7901 xenografts by generation and accumulation of ROS in tumors *in vivo* (Chen W. et al., 2016). A novel analog of curcumin EF24 effectively inhibited cell growth and induced apoptosis mainly *via* producing and accumulating ROS in colorectal cancer HCT-116, SW-620, and HT-29 cells (He et al., 2016). Another analog of curcumin, WZ26, also displayed potent effect on cell growth inhibition with ROS overproduction in gastric cancer cells *in vitro* and greater antitumor activity against tumor xenografts of human gastric cancer *in vivo* compared to curcumin (Zou et al., 2016). Curcumin analog WZ35 exhibited potent cytotoxicity and apoptosis induction against colon cancer CT26 cells* in vitro* and strongly inhibited the tumor growth of CT26 xenografts *in vivo via* ROS mediated signaling pathway (Zhang J. et al., 2017). Other studies also showed that WZ35 could strongly inhibit cell proliferation and induce apoptosis *via* ROS overproduction in prostate cancer RM-1 and DU145 cells *in vitro* and significantly suppress the tumor growth by inducing apoptosis with increased ROS accumulation in the tumor tissues of RM-1 homografts *in vivo* (Chen et al., 2017). Additional studies further showed that WZ35 markedly inhibited the cell proliferation, migration, and invasion with ROS overproduction in liver cancer HCCLM3, HepG2, and Huh-7 cells *in vitro* and exhibited great ability to prevent metastasis of HCCLM3 tumor *in vivo* (Wang L. et al., 2018). L48H37, another novel analog of curcumin, could inhibit cell proliferation and colony formation by accumulation of ROS in human lung cancer cells *in vitro* and suppressed the tumor growth of lung cancer xenografts without host toxicity *in vivo* (Feng et al., 2017). MAC CA10, a new allylated monocarbonyl analog of curcumin, could selectively inhibit cell proliferation and induce apoptosis *via* activating ROS-mediated signaling pathway to generate ROS in gastric cancer cells *in vitro*, and the anticancer activity of MAC CA10 was further demonstrated in an animal model of tumor xenografts *in vivo* (Rajamanickam et al., 2017).

Development of the carriers such as k-carrageenan loading with anticancer drug is a good strategy to increase the anticancer activity. Studies have shown that k-carrageenan loading with curcumin exhibited potent cytotoxicity *via* inducing apoptosis by ROS overproduction in lung cancer A549 cells (Sathuvan et al., 2017). Wang J. et al. (2016) developed the curcumin-loaded and calcium-doped dendritic mesoporous silica nanoparticles modified with folic acid (Cur-Ca@DMSNs-FA) with good solubility and biocompatibility compared to free curcumin, and it effectively inhibited cell growth and induced apoptosis *via* increasing intracellular ROS generation in breast cancer MCF-7 cells.

The dried root of *Lithospermum erythrorhizon* as a TCM has been used for the treatment of burns, sore throat, macular eruptions, carbuncles, and measles for a long time in China (Chen et al., 2002). Report has shown that shikonin, a naphthoquinone derived from the roots of *L. erythrorhizon*, could induce apoptosis through overproduction of ROS in human glioma U87MG and Hs683 cells (Yang J. T. et al., 2014). Studies by Kim et al. (2017) also showed that shikonin could induce cell growth inhibition and apoptosis in human lung cancer A549 cells *in vitro* and inhibit the growth of A549 tumor xenografts *in vivo* through overproduction of intracellular ROS. In addition, shikonin inhibited cell growth and induced ROS-mediated apoptosis by targeting cytosolic thioredoxin reductase in human leukemia HL-60 cells (Duan et al., 2014a). Numerous studies have further shown that shikonin could inhibit cell proliferation and induce apoptosis in multiple human cancer cells including colon cancer SNU-407, liver cancer Hep-G2, breast cancer MDA-MB-231, and endometrioid endometrial cancer Ishikawa, HEC-1A, KLE, and RL95-2 cells (Yingkun et al., 2010; Huang and Hu, 2018; Wen et al., 2018; Han et al., 2019). Additional studies showed that shikonin could inhibit cell growth and induce apoptosis against liver cancer Huh-7 and BEL7402 cells *in vitro* and inhibit the tumor growth of liver cancer xenografts *in vivo via* overproduction of ROS, while ROS scavengers could completely inhibit the anticancer activity of shikonin (Gong and Li, 2011). Shikonin also markedly inhibited the cell proliferation and induced apoptosis *via* enhancing ROS generation against gefitinib-resistant lung cancer H1650 and H1975 cells (Li et al., 2017). Furthermore, shikonin could inhibit the migration and invasion of human breast cancer MDA-MB231 cells (Wei et al., 2013), indicating that shikonin may be effective for the treatment of breast cancer metastasis. Huang et al. (2018) synthesized 45 sulfur-containing shikonin oxime derivatives, and studies have shown that these compounds had more efficacy in inhibiting the cell proliferation and inducing apoptosis against multiple human cancer cells such as breast cancer MCF-7, gastric cancer MGC-803, liver cancer Bel7402, and colon cancer HCT-15 cells than shikonin, with the IC_50_ of 0.27–9.23 μM without noticeable cytotoxicity to normal human skin fibroblast HSF cells.

*Pl. abalonus* (Agaricaceae) is an edible mushroom and its polysaccharides are widely used for dietary supplement. Studies have shown that the polysaccharides isolated and extracted from *Pl. abalonus* exhibited antioxidant and antitumor activities (Ren et al., 2015). Additional studies also showed that the active fractionation (PAP-3) of crude polysaccharides isolated from *Pl. abalonus* could significantly inhibit cell proliferation and induce apoptosis *via* intracellular overproduction of ROS in human breast cancer MCF-7 cells (Shi et al., 2013).

*Isodon rubescens* (Hemsl). H. Hara (Lamiaceae), a popular TCM widely used as a supplement in China, has the functions of antimicrobial, antioxidation, anti-inflammation, and anticancer (Schwarz et al., 2003; Sun et al., 2006). Jaridonin, a novel diterpenoid isolated and extracted from *I. rubescens* (Hemsl). H. Hara could inhibit cell growth and induce apoptosis *via* ROS-mediated pathway in three human esophageal cancer cell lines EC109, EC9706, and EC1 (Ma et al., 2013).

Longikaurin A, an ent-kaurane diterpenoid, was isolated and extracted from *Rabdosia ternifolia* (D. Don) H. Hara (Labiatae), which has been used as a TCM. Studies have shown that longikaurin A could inhibit cell growth, induce apoptosis and G2/M cell cycle arrest, and overproduce ROS in liver cancer SMMC-7721 cells *in vitro* and inhibited the growth of SMMC-7721 tumor xenografts *in vivo via* the regulation of ROS/JNK/c-Jun pathway (Liao et al., 2014). Longikaurin A also exhibited potent cytotoxicity against esophageal cancer KYSE-30 cells through apoptosis induction and ROS generation *in vitro* and suppressed the growth of KYSE-30 tumor xenografts *in vivo* (Che et al., 2017). Additional studies further showed that longikaurin A could inhibit cell proliferation and induce apoptosis and cell cycle arrest in nasopharyngeal cancer CNE2 cells *in vitro*, and attenuate the growth of CNE2 tumor xenografts *in vivo* (Zou et al., 2013).

**Figure 5:**
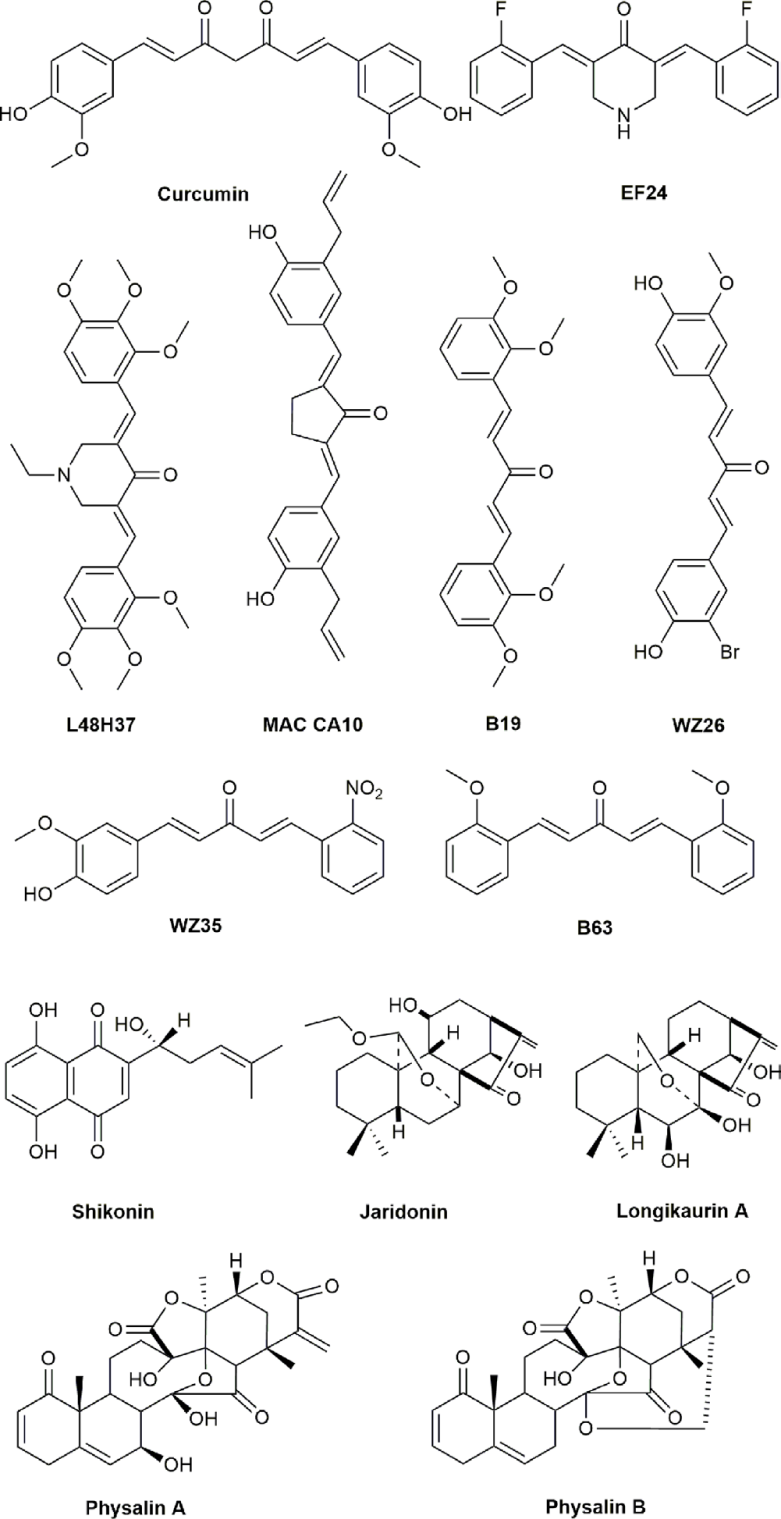
Chemical structures of active compounds derived from anticancer TCMs that primarily activate reactive oxygen species (ROS).

*Physalisalkekengi L.* var. *franchetii* (Mast). Makino (Jindenglong) is a commonly used TCM for the treatment of sore throat, cough, excessive phlegm, dysuria, pemphigus, pharyngitis, eczema, and jaundice in China for over thousands of years (Li A. et al., 2018). Physalin A, a bioactive withanolide isolated and extracted from *Ph. L.* var. *franchetii* (Mast). Makino, could inhibit cell proliferation and induce apoptosis *via* overproduction of ROS in human melanoma A375-S2 cells (He et al., 2013). Physalin A also inhibited cell growth and induced apoptosis and G2/M cell cycle arrest through increasing ROS production in human lung cancer A549 cells (Kang et al., 2016). Physalin B was also isolated and extracted from *Ph. L.* var. *franchetii* (Mast). Makino and significantly inhibited cell growth and induced apoptosis by increasing mitochondrial ROS production in human colon cancer HCT-116 cells (Ma Y. M. et al., 2015). Recent studies further showed that Physalin B displayed the ability to inhibit cell proliferation and induce apoptosis in human breast cancer MCF-7 and lung cancer A549 cells (Wang A. et al., 2018; Cao et al., 2019). The chemical structures of active compounds derived from anticancer TCMs that primarily activate ROS are illustrated in **Figure 5**.

## Anticancer TCMs Through Targeting Pi3k/Akt/Mtor Pathway

PI3K/Akt/mTOR pathway is activated by transmembrane tyrosine kinase growth factor receptors and plays the key role in cell cycle, survival, proliferation, energy metabolism, and apoptosis (Knuefermann et al., 2003; Hassan et al., 2013). The pathway is closely related to other apoptotic pathways and is inhibited by the negative regulator of the pathway phosphatase and tensin homolog (PTEN) (Lim et al., 2015). Furthermore, the activation of the PI3K/Akt/mTOR pathway has been implicated in the pathogenesis and chemoresistance of cancer (Degtyarev et al., 2008; Lim et al., 2015). Inhibition of Akt induced cell death is also associated with autophagy (Degtyarev et al., 2008). Some active compounds derived from anticancer TCMs such as Silybin (Silibinin), Matrine, MASM, WM130, and YYJ18 exert their anticancer activity by mainly inhibiting the PI3K/Akt/mTOR pathway.

*Silybum marianum* (Milk thistle) has been used as a TCM for the treatment of cancer, liver disease, and amanita phalloides poisoning in China (Rainone, 2005). Silybin is a biologically active flavonolignan isolated and extracted from the seeds of *Si. marianum* and contains approximately 50% to 70% of silymarin (Biedermann et al., 2014; Bijak et al., 2017). Silybin could inhibit cell growth and induce apoptosis by inhibiting PI3K/Akt-mTOR signaling pathway in various cancer cells including liver cancer Hep3B, cervical cancer HeLa, renal cancer 769-P, 786-O, OS-RC-2 and ACHN, glioma U87, and multiple myeloma U266 cells (García-Maceira and Mateo, 2009; Ma Z. et al., 2015; Zhang M. et al., 2015; Feng et al., 2016). Silybin also inhibited cell growth and induced apoptosis in breast cancer MCF-7 and SKBR3, lung cancer A549, colon cancer HT-29, and pancreatic cancer Panc-1, AsPC-1, and BxPC-3 cells (Ge et al., 2011; Akhtar et al., 2014; Liang et al., 2014; Yousefi et al., 2014; Jiang et al., 2015). In addition, silybin could enhance the anticancer activity of sorafenib, gefitinib, paclitaxel, and temozolomide against liver cancer SNU761 and Huh-BAT, ovarian cancer SKOV-3, and glioma LN229 cells (Elhag et al., 2015; Gu et al., 2015; Pashaei-Asl et al., 2018). Furthermore, clinical study showed that the combination of silybin and regorafenib enhanced therapeutic efficacy with prolonging overall median survival compared to regorafenib and silybin alone *via* inhibiting PI3K/AKT/mTOR pathway in patients with metastatic colorectal cancer (Belli et al., 2017).

**Figure 6:**
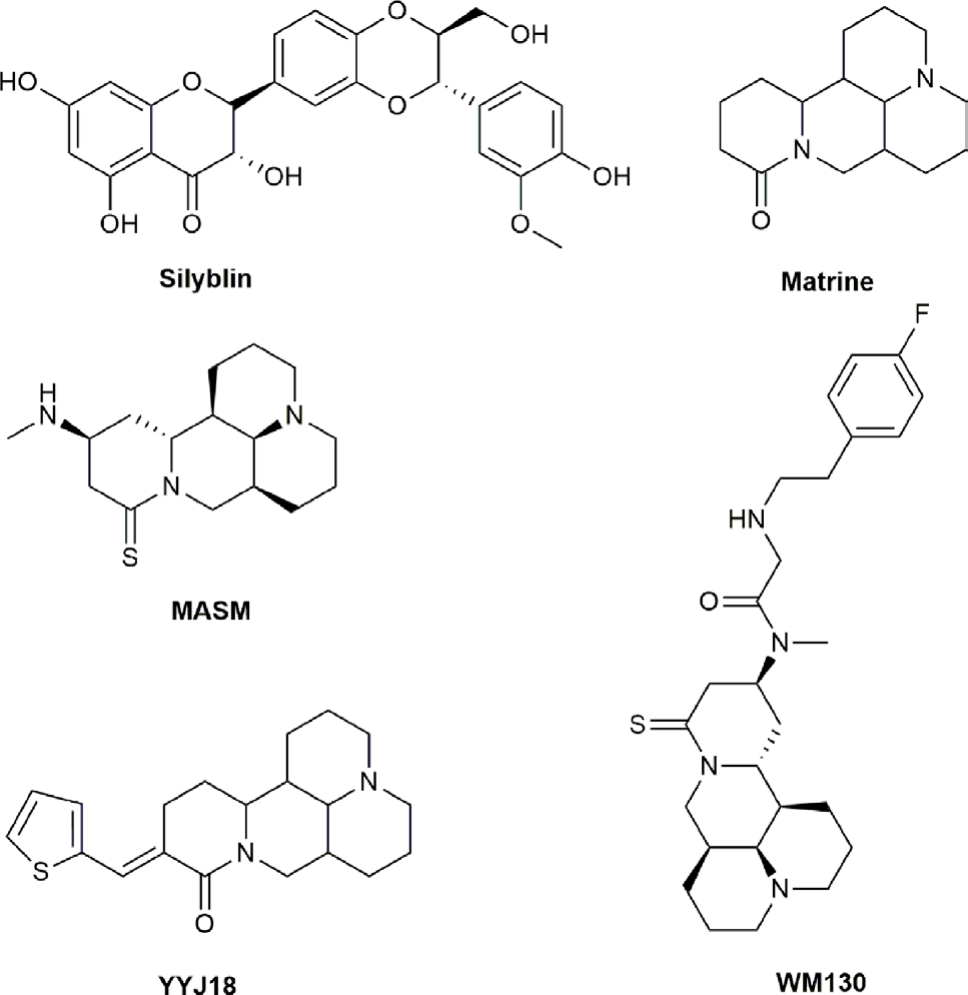
Chemical structures of active compounds derived from anticancer TCMs that primarily target PI3K/AKT/mTOR pathway.

*Sophora flavescens* (SF) is a TCM for the treatment of inflammatory diseases and cancer in China (Sun et al., 2007; Jin et al., 2010). Matrine is an alkaloid isolated and extracted from SF. Recent studies showed that matrine inhibited cell growth, induced apoptosis, and reversed multidrug resistance through inhibiting PI3K/AKT pathway when it was used alone or in combination with other anticancer agents in human breast, prostate, and bladder cancer cells (Liao et al., 2017; Li Q. et al., 2018a; Zhou et al., 2018). The structure analysis revealed that matrine contains a variety of components in which the carbon–carbon double bond at 14′ of its skeleton is critical for anticancer activity (Jiang et al., 2017). Studies have shown that MASM (6aS, 10S, 11aR, 11bR, 11cS)-10-methylamino-dodecahydro-3a,7a-diazabenzo (de), a novel derivative of matrine, markedly inhibited cell growth and induced apoptosis against human liver cancer Hep3B and Huh-7 cells *in vitro* and significantly suppressed the tumor growth of Huh-7 xenograft *in vivo* through inhibiting the PI3K/AKT/mTOR pathway (Liu Y. et al., 2017). Another novel matrine derivative WM130 (C_30_N_4_H_40_SO_5_F) also significantly inhibited cell growth, migration, and invasion, and obviously induced apoptosis in liver cancer Huh-7 cells *in vitro* and markedly suppressed the tumor growth of Huh-7 xenografts *in vivo* through targeting PTEN/Akt pathway (Qian et al., 2015). YYJ18 (14-Thienyl methylene matrine), also a novel matrine derivative, exhibited potent inhibitory effects and apoptosis induction mediated by PI3K/Akt and MAPK pathways in human nasopharyngeal cancer CNE1, CNE2, and HONE1 cells (Xie M. et al., 2014). The chemical structures of active compounds derived from anticancer TCMs that primarily target the PI3K/AKT/mTOR pathway are illustrated in **Figure 6**.

## Anticancer TCMs Through Targeting Jak-Stat3 Signal Pathway

The signaling pathway of Janus kinase (Jak)-signal transducer and STAT3 is constitutively activated and abnormally expressed in cancer cells and plays critical roles in cell survival and apoptosis (Huang, 2007). Studies have shown that p-STAT3, the activated form of STAT3, is elevated in various types of cancer, and it is a marker of poor prognosis for colorectal cancer patients (Kusaba et al., 2006). STAT3 induces the downstream genes encoding for cell cycle regulators, anti-apoptotic proteins, and angiogenic factors to participate in cancer development and progression (Al Zaid Siddiquee and Turkson, 2008). Therefore, Jak-STAT3 signaling pathway represents a valid target for anticancer drugs and effective block of its activation could be a promising strategy for discovery and development of novel anticancer agents in cancer therapy. Some active compounds derived from anticancer TCMs such as cucurbitacin B, DACE, artemisinin, dihydroartemisinin (DHA), BDL301, ursolic acid, and 5,7-dihydroxyflavone could suppress the proliferation and induce apoptosis primarily *via* effectively inhibiting Jak-STAT3 signaling pathway in cancer cells.

Cucurbitacin B, a tetracyclic triterpenoid, was isolated and extracted from the members of cucurbitaceae family such as cucumbers, pumpkins, and gourds (Cai et al., 2015). Studies have demonstrated that cucurbitacin B alone or in combination with other chemotherapeutic agents significantly inhibited cell growth and induced apoptosis by targeting the JAK/STAT3 pathway in various human cancer cells including gastric cancer MKN-45, pancreatic cancer Panc-1, MiaPaCa-2, and PL45, neuroblastoma SH-SY5Y, and osteosarcoma U-2 OS cells (Thoennissen et al., 2009; Zheng Q. et al., 2014; Xie et al., 2016; Zhang Z. R. et al., 2017). Furthermore, DACE, a novel semisynthetic derivative of cucurbitacin B by structure modification of the parental natural compound, exhibited potent inhibitory effects on cell proliferation and apoptosis induction against lung cancer A549 cells *in vitro* and markedly inhibited the growth of c-RAF-induced lung tumors by improving the solubility and bioavailability *in vivo via* targeting STAT3, AkT, and ERK signaling pathways (Silva et al., 2015).

*Artemisia annua* L. has been used as a TCM to treat fever in China for over 2,000 years. Artemisinin, a natural product isolated and extracted from *A. annua* L. in the 1970s in China, is an effective drug for the treatment of resistant malaria (Tu, 2016). The 2015 Nobel Prize in Physiology or Medicine was awarded to Professor Youyou Tu for her major contributions to the discovery of artemisinin (Tu, 2016). In addition, artemisinin possesses various biological and pharmacological properties such as anti-schistosomiasis, antivirus, and anticancer (Saeed et al., 2016; Efferth, 2018; Zhang Y. et al., 2018). Artemisinin exhibited the ability for selectively inhibiting cancer cells but only minimal cytotoxicity to normal cells (Li X. et al., 2018). Most notably, DHA, a derivative of artemisinin with better water solubility, has shown potent effects on cell growth inhibition and apoptosis induction by significantly inhibiting Jak2/STAT3 signaling activation and downstream target proteins in human head and neck cancer FaDu, liver cancer Hep-G2, colon cancer HCT-116, and tongue cancer Cal-27 cells (Jia et al., 2016; Wang D. et al., 2017). Moreover, a study showed that DHA at the concentration of 20 µM induced transient activation of JNK in human umbilical vein endothelial cells (Dong et al., 2016).

BDL301 is a formula of TCM used for the treatment of inflammatory diseases for over hundreds of years. Studies showed that BDL301 could significantly inhibit cell growth and induced apoptosis in colorectal cancer CT-26 and HCT-116 cells *in vitro* and markedly suppress the growth of CT-26 tumors *in vivo via* inhibiting STAT3 signaling pathway (Chu et al., 2015).

*Eriobotrya japonica* (Thunb). Lindl (Rosaceae) and *Ziziphus jujuba* Miller (Rhamnaceae) are TCMs used as supplements for cancer treatment in China. Ursolic acid (UA), a small molecule pentacyclin triterpene isolated and extracted from *E. japonica* (Thunb). Lindl and *Z. jujuba* Miller, inhibited the cell growth and induced apoptosis by targeting the JNK, PI3K/Akt, and NF-κB pathways in human pancreatic cancer Panc-1, MIA PaCa-2, and Capan-1 cells (Li J. et al., 2012). UA also could inhibit cell proliferation and induce apoptosis in human liver cancer SK-Hep-1 cells and the anticancer effects were regulated by the JNK/MAPK, PI3K/Akt, and p38 signaling pathways (Chuang et al., 2016). Additional studies further showed that UA significantly inhibited cell proliferation and induced apoptosis by activating the pro-apoptotic ASK1/JNK signaling pathway in bladder cancer T24 cells (Zheng Q. Y. et al., 2013).

**Figure 7:**
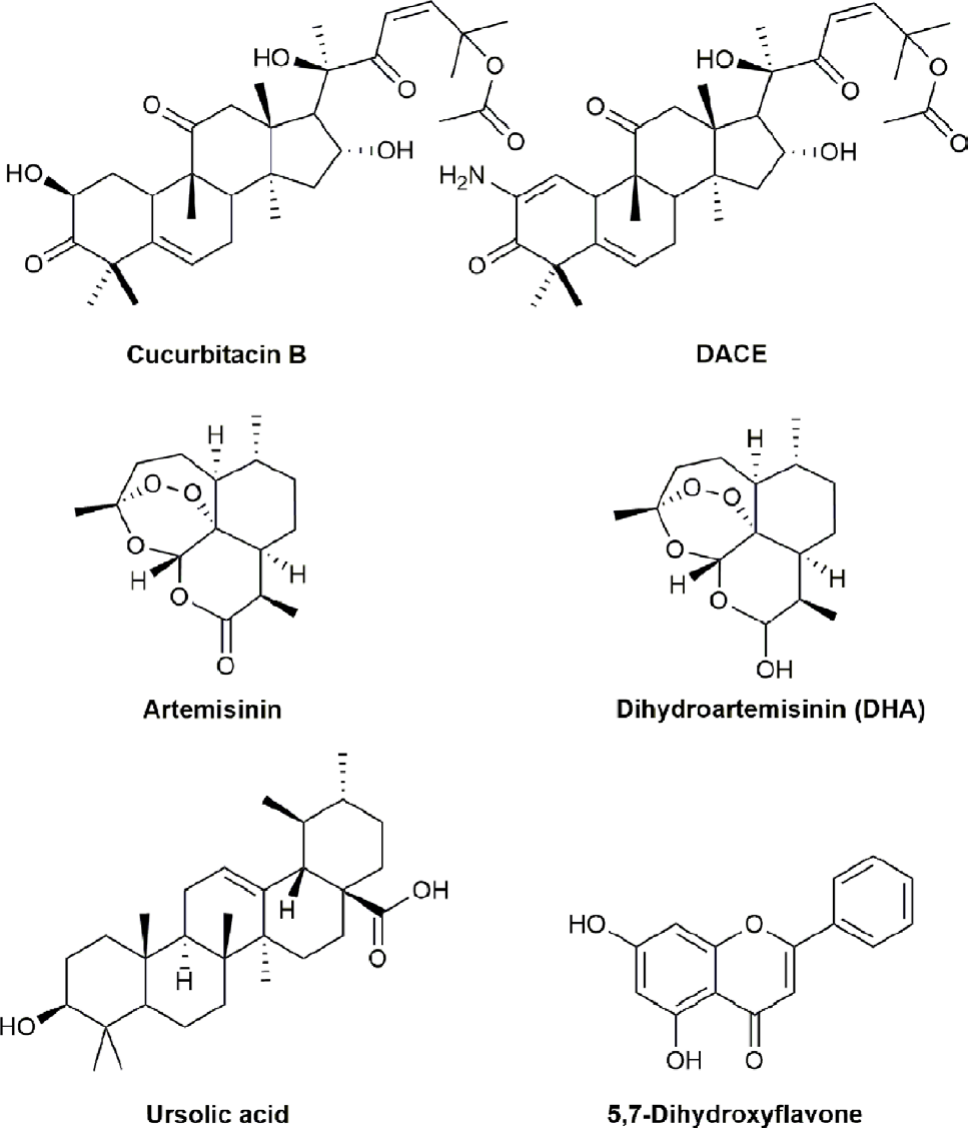
Chemical structures of active compounds derived from anticancer TCMs that primarily target the Janus kinase (Jak)-signal transducer and activator of transcription 3 (STAT3) signaling pathway.

Studies showed that 5,7-dihydroxyflavone, a dietary flavonoid that commonly exists in various plants, inhibited cell viability and induced apoptosis against human liver cancer HepG2 cells with minimal cytotoxicity to normal hepatocyte L-O2 cells *in vitro* and markedly suppressed the growth of HepG2 tumor xenografts *in vivo via* reducing the phosphorylations of Akt and STAT3 (Zhang Z. et al., 2013). The chemical structures of active compounds derived from anticancer TCMs that induce apoptosis by mainly targeting the Jak/STAT3 signaling pathway are illustrated in **Figure 7**.

## Anticancer TCMs Through Targeting Multiple Apoptotic Pathways

Unlike regular pharmaceutical drugs that execute their therapeutic effects on monotarget, TCMs execute their anticancer activity as multitarget molecules by targeting multiple cellular signaling pathways (Faivre et al., 2006). Therefore, targeting multiple cellular signaling pathways is one of the major properties of anticancer TCMs and derived active compounds. Here, we summarize some typical active compounds derived from anticancer TCMs that inhibit cell growth and induce apoptosis by targeting multiple apoptotic signaling pathways in cancer cells including icariside II, osthole (OST), evodiamine (EVO), ginsenosides (compound K, Rh2 and Rk3), extracts of *Brucea javanica*, *B. javanica* oil emulsion injection (Yadanzi^®^), the extracts of *Coix lacryma-jobi* L. seed, Kanglaite (KLT), bufotalin, cinobufacin, bufalin, and cinobufagin.

*Herba epimedii* is a TCM and traditionally used for the relief of stress and fatigue in China for a long time. Icariside II, a flavonoid glycoside isolated and extracted from *H. epimedii*, could inhibit cell proliferation and induce apoptosis by targeting MAPK/ERK, PI3K/Akt, STAT3, Cox-2/PGE2, and β-catenin signaling pathways in multiple cancer cells (Khan et al., 2015). Studies have shown that icariside II could also significantly inhibit the cell growth and induce apoptosis through upregulation of Bax, cleaved caspases-3, -7, -9, and poly (ADP-ribose) polymerasein, as well as downregulation of Bcl-2 in human osteosarcoma U2OS cells (Tang et al., 2017). Other studies also showed that icariside II inhibited the epithelial-mesenchymal transition (EMT), migration, and invasion *via* inhibiting Akt/NF-κB signaling pathway at inflammatory microenvironment induced by TNF-α in human lung cancer A549 and H1299 cells (Song et al., 2017).

*Cnidium monnieri* (L). Cusson exhibits potent anti-allergic, antidermatophytic, antipruritic, antibacterial, antifungal, and anti-osteoporotic activities and is one of the most widely used TCMs for the treatment of various diseases including male impotence, female genital diseases, frigidity, and skin-related diseases in China, Vietnam, and Japan (Li et al., 2015). OST was isolated and extracted from the dried fruit of *Cn. monnieri* (L). Cusson and exhibited significant anticancer activity in different cancer cells *in vitro* and tumor growth inhibition against various animal models of tumor xenografts *in vivo via* targeting multiple apoptotic signaling pathways (Jarząb et al., 2017). For example, OST could inhibit cell proliferation and induce apoptosis by downregulating caspase-3 expression and upregulating the Bax/Bcl-2 ratio in human osteosarcoma MG-63 cells (Ding et al., 2014). OST also could induce apoptosis and inhibit cell proliferation, migration, and invasion by regulating the PTEN/Akt pathway in osteosarcoma MG-63 and SAOS-2 cells (Wang L. et al., 2016). In addition, OST markedly inhibited the cell proliferation and induced apoptosis *via* targeting the PI3K/Akt pathway in intrahepatic cholangiocarcinoma HCCC-9810 and RBE cells (Zhu et al., 2017). Studies further showed that OST could inhibit cell proliferation and induce apoptosis through targeting PI3K/Akt and MAPK signaling pathways in rat glioma C6 cells (Ding et al., 2013) and via regulating apoptotic proteins Bcl-2, Bax, and Caspase 3/9 in ovarian cancer A2780 and OV2008 cells (Jiang G. et al., 2016). Moreover, OST could sensitize cellular apoptosis induced by TRAIL *via* downregulating the expression of cellular FLICE-like inhibitory protein (c-FLIP) in human breast cancer MDA-MB-231, renal cancer Caki, and glioma U251MG cells (Min et al., 2017). However, the clinical application of OST is limited by its poor bioavailability due to insolubility in water. To overcome the problem, OST was successfully loaded into N-caprinoyl-N-trimethyl chitosan (CA-TMC, an amphiphilically modified chitosan derivative) as micelles, and the results showed that OST/CA-TMC micelles exhibited significant anticancer activity with potent cytotoxicity and apoptosis induction in human breast cancer MCF-7 and cervical cancer Hela cells (Hu et al., 2013).

*Evodia rutaecarpa* has been widely used as a TCM for the treatment of pain, aches, vomiting, and dysentery in China for centuries (Li W. et al., 2016). EVO (8,13,13b,14-tetrahydro-14-methylindolo[2’3’-3,4]pyrido[2,1-b]quinazolin-5-[7H]) is a novel quinolone alkaloid isolated and extracted from *E. rutaecarpa* (Gavaraskar et al., 2015). EVO could significantly inhibit the cell growth and enhance apoptosis *via* inducing G2/M cell cycle arrest and targeting the apoptotic, MAPK, and PI3K/Akt pathways in human ovarian cancer HO-8910PM cells (Wei et al., 2016). EVO also significantly inhibited cell proliferation and induced apoptosis through activating JNK and PERK in human ovarian A2780, A2780CP, ES-2, and SKOV-3 cells (Chen T. C. et al., 2016). Additional studies showed that EVO could significantly inhibit the cell growth and induce apoptosis and G2/M cell cycle arrest *via* activating JNK in human colon COLO205 and HT-29 cells (Chien et al., 2014), and targeting mitochondrial and ER pathways in human lung cancer H446 and H1688 cells (Fang et al., 2014). Further studies showed that 24–48 h treatment of EVO at 20–40 μM significant inhibited cell growth and increased apoptosis with the activation of caspases-9, -3, and -8 and DR5 and the alteration of the ratio of Bax/Bcl-2 in human lung cancer A549 and H1299 cells (Mohan et al., 2016). EVO also could inhibit cell proliferation, induce G2/M cell cycle arrest, and promote apoptosis through activating caspases-3, -8, and -9, as well as altering the ratio of Bax/Bcl-2 in human gastric cancer SGC-7901 cells (Yang, L. et al., 2014). In addition, EVO enhanced the efficacy of radiation therapy by significantly inhibiting the cell cycle progression and growth in human gastric cancer BGC-823 cells *in vitro* and markedly suppressing the tumor growth of BGC-823 xenografts *in vivo*, and the anticancer effect of EVO was related to the downregulation of the Her2/AkT/Bcl-2 signaling pathway (Hu C. et al., 2016). Furthermore, EVO alone or in combination with chemotherapeutic agent wortmannin could inhibit cell growth, migration, and EMT and induce apoptosis by decreasing the levels of Bcl-2, phospho-Akt, and matrix metalloproteinases-2 and -9 proteins and increasing the levels of p21 and p53 proteins in thyroid cancer TPC-1 and SW1736 cells (Kim et al., 2018). EVO could also inhibit cell growth, induce apoptosis and cell arrest *via* increasing the levels of phosphorylated Bcl-2 protein, ER stress protein, and protein kinase RNA-like ER kinase in human renal cancer A498 cells *in vitro*, and suppress the growth of A498 tumors *in vivo* (Wu et al., 2016).

*Panax ginseng* C. A. Mey (Araliaceae) is a popular TCM for over 2,000 years and it has buzhongyiqi (补中益气) activity (supplementing the center and boosting energy) as described in Ben Cao Gang Mu (“Compendium of Materia Medica”). Pharmacological studies showed that the extracts of *P. ginseng* C. A. Mey contain many active constituents with numerous beneficial activities including anticancer, antidiabetes, antiaging, antifatigue and antistress as well as pain-relieving, improvement of brain and sexual functions, enhancement of the immune system and liver functions, and so on (Park et al., 2005; Ru et al., 2015). Multiple studies have shown the anticancer activity of *P. ginseng* C. A. Mey extracts preclinically and clinically, and the anticancer effect is mainly attributed to ginsenosides (Cheng et al., 2005; Choi and Choi, 2009; Park et al., 2009; Yi et al., 2009; King and Murphy, 2010; Nag et al., 2012; Duan et al., 2017). Ginseng water extract and its fractions of ginsenoside and polysaccharide inhibited cell growth, enhanced apoptosis, and induced G0/G1 cell cycle arrest by increasing the expressions of cleaved caspase-3 and Bax in human colon cancer HCT-116 cells (King and Murphy, 2010). Ginsenoside Rh2 (a purified ginseng saponin) could inhibit cell proliferation and induce apoptosis *via* increasing the expression of TRAIL-RI (DR4) death receptor and activating caspases-2, -3, and -8 in human lung cancer A549 cells (Cheng et al., 2005). Ginsenoside Rh2 also induced apoptosis by inducing ligand-independent Fas activation in human cervical cancer HeLa cells (Yi et al., 2009). Additional study also showed that ginsenoside metabolites compound K and Rh2 induced apoptosis by targeting p38/MAPK and apoptotic signaling pathways in human astrocytoma CRT-MG cells (Choi and Choi, 2009). Ginsenoside Rk3 inhibited cell growth, enhanced apoptosis, and induced G1 cell cycle arrest by activating caspases-3, -8, and -9, upregulating the expressions of Bax and P21, and downregulating the expressions of Bcl-2, cyclin D1, and CDK4 in human lung cancer H460 and A549 cells *in vitro* and significantly inhibited the tumor growth of H460 xenografts without obvious toxicity to the host *in vivo* (Duan et al., 2017). In addition, the extract of red ginseng could inhibit cell proliferation and induce apoptosis by activating caspase-3, downregulating antiapoptotic Bcl-2 and Bcl-X(L), and decreasing telomerase activity in human leukemia U937 cells (Park et al., 2009). In a review article by Nag et al. (2012), it was discussed that ginsenosides (saponins and the main active principals of ginseng) display their anticancer effects against various cancer cells *in vitro* and tumors *in vivo via* modulation of different cellular signaling pathways such as protein kinases (JNK, Akt, and AMPK), growth factors (EGFR and VEGF), cell proliferation (CDKs and cyclins), death mediators (Bcl-2, Bcl-xL, XIAP, caspases, and death receptors), inflammatory response molecules (NF-κB and COX-2), oncogenes (c-myc and MDM2), and tumor suppressors (p53 and p21).

*B. javanica* (L). Merr. (Simaroubaceae) is a TCM used for the treatment of corns and warts in China as described in the “Compendium of Materia Medica” published in the 16th century. Studies have shown that *B. javanica* oil inhibited the cell growth and induced apoptosis by significantly upregulating the expressions of caspases-3 and -9, while inhibiting the expressions of COX-2 and NF-kB in bladder cancer T24 cells (Lou et al., 2010). The seed oil of *B. javanica* also induced cytotoxicity and apoptosis *via* targeting apoptotic signaling pathway in human AML U937 and HL-60 cells *in vitro* and significantly inhibiting the growth of U937 tumor xenografts *in vivo* (Zhang et al., 2011). The extract of *B. javanica* fruit inhibited cell growth and induced apoptosis by targeting apoptotic signaling pathway in pancreatic cancer PANC-1, SW1990 and CAPAN-1 cells (Lau et al., 2008). Studies also demonstrated that *B. javanica* oil emulsion could induce apoptosis and inhibit autophagy in colon cancer HCT-116 cells (Yan et al., 2015) and induce apoptosis and G0/G1 cell cycle arrest by downregulating the expressions of p53 and Bcl-2 in liver cancer SMMC-7721 cells (Ma and Zhang, 2004). *B. javanica* oil emulsion also could significantly inhibit cell proliferation and enhance the radiosensitivity by inducing apoptosis and inhibiting hypoxia-inducible factor 1α (HIF-1α) in esophageal cancer ECA109 cells *in vitro* and ECA109 tumors *in vivo* (Pan et al., 2018). A phase II clinical study showed that *B. javanica* oil emulsion injection (Yadanzi^®^) combined with chemotherapeutic agents such as pemetrexed and platinum could increase anticancer activity and decrease toxicity compared to chemotherapy alone in 58 patients with advanced lung cancer (Lu et al., 2013). Another phase II clinical study also showed that the combination of Yadanzi^®^ injection with chemotherapy was an effective and safe regimen with 85.3% overall response rate in 75 patients with advanced gastric cancer (Liu et al., 2013). A meta-analysis of 2,234 patients with advanced non-small-cell lung cancer (NSCLC) by Xu et al. (2016) showed that *B. javanica* oil emulsion injection enhanced the efficacy and decreased chemotherapy-induced toxicities including nausea and leukopenia and therefore improved patients’ quality of life. Yadanzi^®^, the oil emulsion from the seed of *B. javanica* (L). Merr., has been approved as an adjunctive therapy for the treatment of patients with lung, prostate, or gastrointestinal cancers in China by the China Food and Drug Administration (CFDA).

*C. lacryma-jobi* L. (Gramineae) as a TCM has been widely used for the treatment of warts, chapped skin, inflammation, rheumatism, and neuralgia in China for over 2,000 years (Kuo et al., 2012). Studies have shown that *C. lacryma-jobi* L. possesses multiple activities including anticancer (Chang et al., 2003; Hung and Chang, 2003; Shih et al., 2004; Lee et al., 2008; Chung et al., 2011b);, anti-mutagenicity (Chen et al., 2011), anti-proliferation (Kuo et al., 2001), antioxidant (Kuo et al., 2001; Yu et al., 2011), anti-inflammation (Huang et al., 2009), anti-ulcer (Chung et al., 2011a), antimicrobial (Ishiguro et al., 1993; Nguta et al., 2015), antiallergy (Chen et al., 2010), and so on. Reports showed that a methanolic extract of *C. lacryma-jobi* L. seed inhibited cell proliferation, enhanced apoptosis, and induced cell cycle arrest in human lung cancer A549 cells *in vitro* and significantly inhibited the tumor growth of A549 xenografts *in vivo via* suppressing the expression of COX-2 (Chang et al., 2003; Hung and Chang, 2003). Several compounds isolated and extracted from the bioactive subfraction of *C. lacryma-jobi* L. bran ethanolic extract significantly inhibited cell proliferation against human breast cancer MCF-7, MDA-MB-231, and T-47D cells (Chung et al., 2011b). Five active compounds isolated and extracted from *C. lacryma-jobi* L. exhibited potent cytotoxicity against human lung cancer A549 and colorectal cancer HT-29 and COLO 205 cells with the IC_50_ values of 28.6–72.6 mg/ml (Lee et al., 2008). The hexane fraction of *C. lacryma-jobi* L. ethanolic extracts could significantly inhibit cell growth and synergistically enhance the anticancer efficacy of doxorubicin by inducing apoptosis and reducing the expression of P-gp in human uterine sarcoma parent MES-SA and doxorubicin-resistant MES-SA/Dx5 cells (Chang et al., 2018). Kanglaite (KLT) injection was isolated and extracted from the seed of *C. lacryma-jobi* L. and it was approved as a new anticancer drug by the CFDA in 1995 and the Federal Service for Surveillance in Healthcare (*Roszdravnadzor*) of **Russia** in 2003. Studies have shown that KLT injection exhibited significant anticancer activity against various cancer cells *in vitro* and tumors *in vivo* (Wang et al., 1999; Guo et al., 2001; Wu L. Q. et al., 2004; Pan et al., 2012; Liu Y. et al., 2014). Mechanistic studies of KLT showed that KLT could inhibit cell growth and induce apoptosis by upregulating the expressions of p53, Fas, caspase-3, proliferating cell nuclear antigen (PCNA), and P21^WAFI/CIPI^, while downregulating the expressions of cyclins A, E1, and F in cancer cells (Wang et al., 1999; Guo et al., 2001; Li and Shi, 2002; Bao et al., 2004; Yuan et al., 2004). Additional studies demonstrated that KLT exhibited immunomodulatory activity by reducing the levels of the cytokines IFN-γ and IL-2, rescuing the levels of CD4+ T cells, and enhancing the cytotoxic activities of natural killer (NK) and CD8+ T cells in the serum of nude mice bearing human liver cancer HepG2 xenografts and stimulated the immune response by increasing the number of T cells and NK cells in the blood of patients with liver cancer (Huang et al., 2014). KLT enhanced the anticancer activity of Taxol *via* inhibiting NF-κB and upregulating connexin 43 in colorectal cancer HCT106, HCT116, LoVo, and CT26 cells *in vitro* and CT26 tumors *in vivo* (Wang Y. et al., 2017). Clinical studies and meta-analyses showed that the combination of KLT injection and chemotherapeutic agents improved the response rate, symptoms, and quality of life, and reduced the incidence of chemotherapy-induced side effects compared to chemotherapy alone in patients with primary NSCLC (Liu X. et al., 2008; Liu X. et al., 2014), unresectable hepatocellular carcinoma (Fu et al., 2014), gastric cancer (Zhan et al., 2012), or pancreatic cancer (Zhang D. et al., 2017).

*Venenum bufonis*, a well-known TCM, is isolated and extracted from the dried skin of toad and has the function of “解毒消肿, cleaning toxics and annihilating swelling” as recorded by Bencao Gangmu (“Compendium of Materia Medica”); it has been commonly used for the treatment of cardiotonic, diuretic, anodyne, cancer, and inflammatory diseases in China (Qi et al., 2011b; Qi et al., 2014). Cinobufacin (huachansu), an injectable preparation of *V. bufonis*, was clinically developed in China for the treatment of cancer in the 1990s. Studies showed that cinobufacin significantly induced apoptosis *via* caspase-mediated apoptotic pathway against liver cancer cells (Qi et al., 2010). Clinical study of 76 patients with gastric cancer showed that the anticancer efficacy was markedly increased and drug-induced toxicity was significantly decreased when cinobufacin was combined with different chemotherapeutic drugs such as docetaxel, cisplatin, or 5-fluorouracil and therefore improved the quality life of the patients (Gong et al., 2010).

Bufotalin was isolated and extracted from *V. bufonis* and exhibited potent cytotoxicity against human liver cancer HepG2 cells and doxorubicin-induced multidrug resistant R-HepG2 cells; the effect of bufotalin on cell growth inhibition was stronger against R-HepG2 cells than that of the parent HepG2 cells *in vitro* and significantly inhibited the tumor growth of R-HepG2 xenografts with minimal host toxicity *in vivo* (Zhang D.M. et al., 2012). Further studies indicated that the effect of bufotalin on apoptosis induction was associated with multiple mechanisms including caspases-9 and -3 activation, PARP cleavage, Bax/Bcl-2 ratio alteration, mitochondrial membrane potential decrease, intracellular calcium level and ROS production increase, and Akt expression and phosphorylation inhibition (Zhang D.M. et al., 2012).

**Figure 8:**
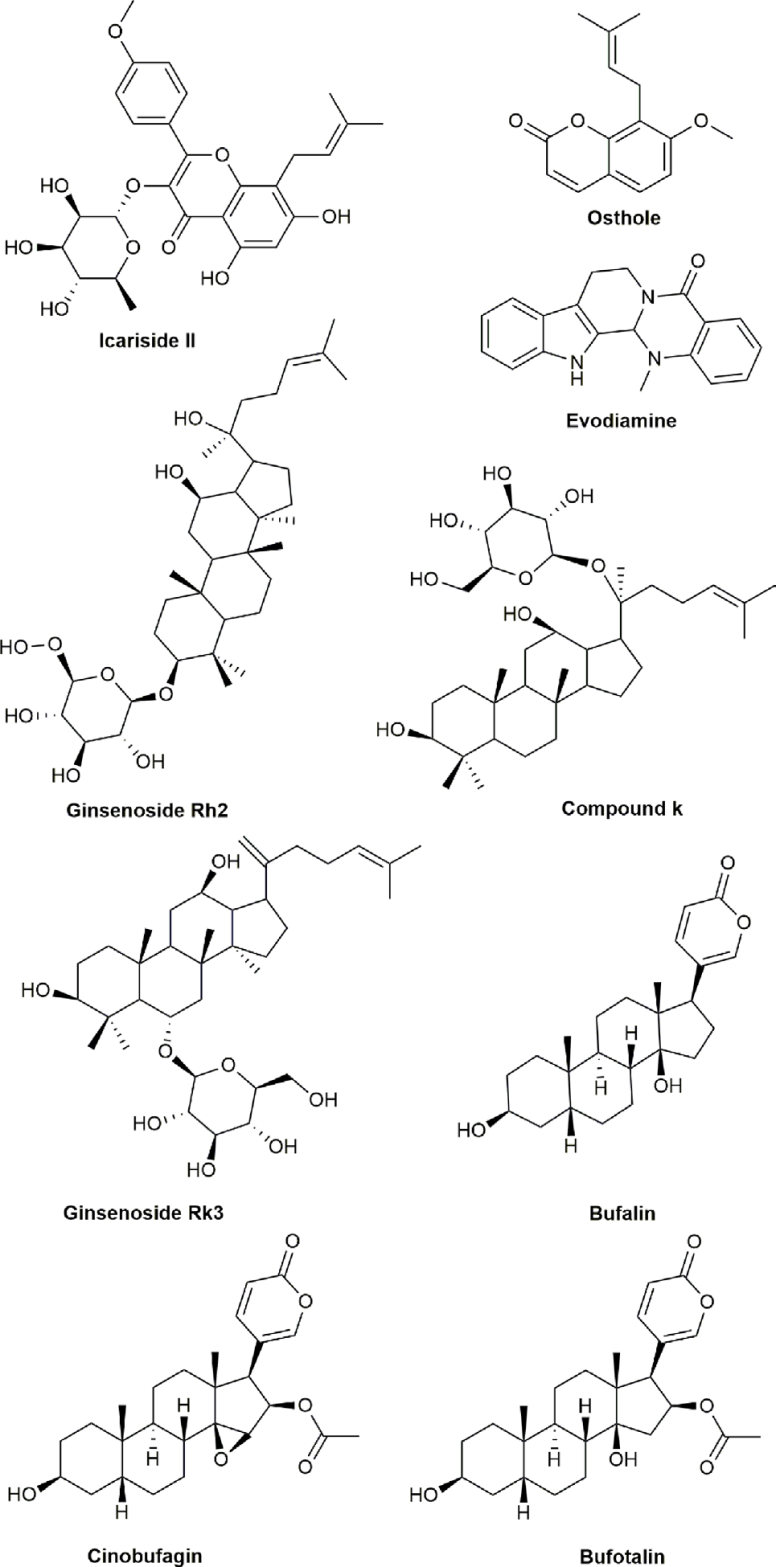
Chemical structures of active compounds derived from anticancer TCMs that target multiple apoptotic signaling pathways.

Bufalin and cinobufagin are cardiotonic steroids and the major bioactive components isolated and extracted from the skin of *V. bufonis* (Jiang et al., 2010; Qi et al., 2011a). Numerous studies revealed that bufalin significantly inhibited cell growth and enhanced apoptosis *via* targeting multiple signaling pathways such as Bcl-2/Bax, Cyt-c, caspases-3, -8, -9, and -10, Fas, PARP, PI3K/Akt, JNK, Erk MAP kinase, ROS, Cox-2, p53, p21^WAF1^, cyclin D1, and histone acetylation in various cancer cells including lung, liver, colorectal, gastric, prostate, bladder cancers, as well as malignant melanoma, osteosarcoma, and leukemia (Yu et al., 2008; Amano et al., 2009; Li et al., 2009; Qi et al., 2011a; Xie et al., 2011; Hong and Choi, 2012; Hsiao et al., 2012; Wang H. et al., 2016; Lee et al., 2017). Bufalin also could enhance the anticancer efficacy of kinase inhibitor sorafenib and Hedgehog signaling pathway inhibitors (GANT61 and cyclopamine) against human liver cancer PLC/PRF/5, SMMC7721, and LM3 cells by inhibition of the PI3K/AkT pathway, downregulation of VEGF, MMP-2, MMP-9, and β-catenin, and upregulation of E-cadherin (Sheng et al., 2016; Wang H et al., 2018). Cinobufagin could inhibit cell growth and induce apoptosis by targeting apoptotic modulators including Bax, Cyt-c, and caspases (-3, -8, and -9) in prostate cancer LNCaP, DU145, and PC3 cells (Yu et al., 2008) and liver cancer HepG2 cells (Qi et al., 2011a). The chemical structures of active compounds derived from anticancer TCMs that target multiple apoptotic signaling pathways are illustrated in **Figure 8**.

## Conclusion

Cancer is one of the leading causes of death in the world. Chemotherapy is one of the classic and important types of cancer treatment, particularly for later stage of cancer with metastasis. Recently, great progress has been made with more and more new therapeutic drugs including chemotherapy, targeted therapy, and immunotherapy being approved for cancer therapy due to the advances in cellular and molecular biology, immunology, and genomics leading to discovery of many novel oncogenes, tumor suppressor genes, and immunologic and therapeutic targets. However, drug-induced toxicities and resistance limit the efficacy and clinical applications of therapeutic drugs. Therefore, the discovery and development of novel anticancer agents are urgently needed to improve efficacy and reduce toxicity.

Apoptosis is one of the key mechanisms of anticancer drugs in cancer therapy and has emerged as an effective target for the discovery and development of novel anticancer agents. Numerous TCMs exhibited excellent anticancer activity by inducing apoptosis in various cancer cells and have been discovered and developed as novel anticancer agents recently. Some active compounds obtained from TCMs have been validated to be effective treatment for patients with various types of cancer clinically. Anticancer TCMs and their derivatives could induce apoptosis *via* targeting one or even multiple cellular signaling pathways including caspase proteases, Bcl-2/Bax, NF-κB, ROS, PI3K/AKT/mTOR, JAK/STAT3, and so on. The anticancer TCMs and their derivatives discussed in the review are summarized in **Table 1** according to their mechanistic action.

**Table 1:** Summary of the therapeutic targets for anticancer traditional Chinese medicines (TCMs) and related derivatives in cellular apoptotic pathway.

Anticancer TCMs and related derivatives	Target
*Cordyceps militaris* (Cordycepin), *Stephania tetrandra* S. Moore (Tetrandrine), *Scutellaria barbata* D. Don, *Crocus sativus* L. (crocin), *Agrimonia pilosa* Ledeb	Caspase
*Prunella vulgaris* (hyperoside), *Rabdosia rubescens* (oridonin), *Solanum incanum* (solamargine), *Solanum lyratum Thunb*, *Trametes robiniophila* Murr (Huaier), *Hedyotis diffusa* Willd, *Garcinia hanburyi* Hook. f. (Gambogic acid), *Carpesium divaricatum* (Telekin)	Bcl-2/Bax
*Tripterygium wilfordii* Hook. f. (Triptolide, Minnelide), *Rubia yunnanensis* (RA-XII, rubiarbonol G), *Scutellaria baicalensis* Georgi (wogonin, baicalein, baicalin), *Lycium chinense* Miller (Cortex lycii radices), *Paeonia lactiflora* Pall (paeoniflorin), *Nigella sativa*	NF-κB
*Curcuma longa* Linn (Curcumin, B63, B19, EF24, WZ26, WZ35, L48H37, MAC CA10), *Lithospermum erythrorhizon* (shikonin), *Pleurotus abalonus* (polysaccharides), *Isodon rubescens* (Hemsl). H. Hara (Jaridonin), *Rabdosia ternifolia* (D. Don) *H. Hara* (Longikaurin A), *Physalis alkekengi L. var. franchetii* (Mast). *Makino* (Physalin A, Physalin B)	ROS
*Silybum marianum* (Silybin), *Sophora flavescens* (Matrine, MASM, WM130, YYJ18).	PI3K/Akt/mTOR
Cucurbitacin B, DACE, *Artemisia annua* L. (artemisinin, dihydroartemisinin), BDL301, *Eriobotrya japonica* (Thunb). Lindl, *Ziziphus jujuba* Miller (Ursolic acid), and 5,7-dihydroxyflavone.	Jak-STAT3
*Herba epimedii* (Icariside II), *Cnidium monnieri* (L). Cusson (Osthole), *Evodia rutaecarpa* (Evodiamine), *Panax ginseng* C. A. Mey (Compound k, Ginsenoside Rh2, Ginsenoside Rk3), *Brucea javanica* (L). Merr (*Brucea javanica* oil emulsion, (Yadanzi®), *Coix lacryma-jobi* L. (Kanglaite), *Venenum bufonis* (cinobufacin, bufotalin, bufalin, cinobufagin).	Multiple signaling pathways

Natural products such as TCMs are important source for discovery and development of novel compounds against cancer. The use of drug substances derived from natural sources to develop novel anticancer drugs has a long tradition in medicine. Recently, active ingredients isolated and extracted from TCMs for cancer treatment have attracted substantial attention. It is well known that anticancer TCMs consist of various active components that can selectively inhibit different cellular signaling pathways and regulate the proliferation, survival, EMT, migration, invasion, metastasis, apoptosis, and microenvironment of cancer cells.

Development of anticancer drugs from TCMs has several advantages including the following: easy to be obtained with unlimited resources, significantly lower cost for development compared to conventional chemotherapeutic drugs, and many of them could be orally administrated. Studies have demonstrated that numerous anticancer agents developed from TCMs have shown significant antitumor efficacy with less toxic/side effects, and less drug resistance compared to clinically commonly used traditional chemotherapeutic agents; many of them exhibited curative effect by preventing recurrence and prolonging survival of patients with cancer, particularly when they are combined with other chemotherapeutic and/or targeted anticancer drugs. However, many obstacles and challenges still remain for the discovery and development of anticancer TCMs such as the following: 1) Poor bioavailability of natural compounds from TCMs. 2) The majority of compounds from anticancer TCMs were investigated only *in vitro*, without *in vivo* and clinical trials to elucidate and validate the full spectrum of anticancer efficacy, side effects, pharmacology, and the further clinical usefulness as anticancer agents. 3) Most studies of the compounds from anticancer TCMs were focused on monomer or active ingredient isolated and extracted from TCMs and lack of TCM theory, which supports synergistic anticancer effect from different components, as demonstrated by TCM extracts that exhibit more potent anticancer efficacy than each individual component alone in many TCM preparations. In order to overcome the obstacles, the following directions for the future may be considered: 1) Synthesis of novel analogues from structural modifications with advanced technologies to improve the bioavailability of TCM and enhance anticancer efficacy and selectivity; 2) utilization of nanoparticles, microspheres, and solid lipid liposomes carries anticancer agents isolated from TCMs to improve their bioavailability and to enhance anticancer activities and decrease the side effects of the drugs by accuracy of drug delivery to particular locations inside the cells or tissues; 3) systematic *in vivo* studies for the agents from anticancer TCMs including animal models of human cancer, pharmacokinetics, pharmacodynamics, and drug metabolism to elucidate anticancer efficacy and side effects, and to determine the appropriate route and schedule for administration; 4) large-scale clinical trials are required for the agents derived from TCMs in the treatment of patients with different types of cancer to validate the anticancer efficacy, side effects, and safety profile; and 5) combination of different agents obtained from anticancer TCMs and/or clinically commonly used conventional chemotherapeutic drugs to synergistically enhance the overall anticancer efficacy in cancer therapy rather than the use of a single agent. Understanding the functions and molecular mechanisms of anticancer TCMs is critical and important for discovery and development of more effective novel anticancer drugs from TCMs. We believe that many more effective or even curative novel anticancer drugs from TCMs will be discovered and developed to enter the market as a potential choice for oncologists in cancer therapy in the near future.

## Author Contributions

SC and XL designed the work. WA, HL, YZ, ML, XL, and SC collected and reviewed the references. WA and HL wrote the first draft. SC wrote and XL reviewed the final version of the manuscript. All authors discussed and contributed to the manuscript.

## Funding

This work was supported by grants from the National Innovative Drug Development Project (2014ZX-091 02043-001), National Natural Science Foundation of China (81302906, 81273550, and 41306157), and Distinguished Professor Research Startup Funding (2015-RCYJ0002) from Southwest Medical University.

## Conflict of Interest Statement

The authors declare that the project was conducted in the absence of any commercial or financial relationships that could be construed as a potential conflict of interest.
